# Influencing immunity: role of extracellular vesicles in tumor immune checkpoint dynamics

**DOI:** 10.1038/s12276-024-01340-w

**Published:** 2024-11-11

**Authors:** Ziyang Ye, Genpeng Li, Jianyong Lei

**Affiliations:** Division of Thyroid Surgery, Department of General Surgery, West China Hospital, Sichuan University, Chengdu, China

**Keywords:** Immunoediting, Tumour biomarkers

## Abstract

Immune checkpoint proteins (ICPs) serve as critical regulators of the immune system, ensuring protection against damage due to overly activated immune responses. However, within the tumor environment, excessive ICP activation weakens antitumor immunity. Despite the development of numerous immune checkpoint blockade (ICB) drugs in recent years, their broad application has been inhibited by uncertainties about their clinical efficacy. A thorough understanding of ICP regulation in the tumor microenvironment is essential for advancing the development of more effective and safer ICB therapies. Extracellular vesicles (EVs), which are pivotal mediators of cell–cell communication, have been extensively studied and found to play key roles in the functionality of ICPs. Nonetheless, a comprehensive review summarizing the current knowledge about the crosstalk between EVs and ICPs in the tumor environment is lacking. In this review, we summarize the interactions between EVs and several widely studied ICPs as well as their potential clinical implications, providing a theoretical basis for further investigation of EV-related ICB therapeutic approaches.

## Extracellular Vesicles Influence Immune Checkpoints in Tumors

In 1991, the finding of CTLA4 led to extensive studies on immune checkpoint proteins, key in controlling immune reactions. This review explores the role of extracellular vesicles—small particles released by cells carrying proteins, RNA, and lipids, affecting various cell functions—in immune checkpoint control. The research focuses on how EVs influence immune checkpoints, especially in cancer, where they can change immune cell function and affect therapy results. The authors aim to discover new treatment strategies by studying the interaction between EVs and immune checkpoints. This detailed review highlights the potential of targeting EV-related pathways to improve cancer treatment effectiveness and safety. The results emphasize the need for more research into EVs and immune checkpoints, potentially leading to significant progress in cancer treatment.

This summary was initially drafted using artificial intelligence, then revised and fact-checked by the author.

## Introduction

The discovery of CTLA4 as a pivotal inhibitor of the immune response in 1991 marked the beginning of extensive research in immune checkpoint proteins (ICPs)^1^, leading to the identification of various members of this large family, which includes inhibitors such as programmed death-ligand 1 (PD-L1), cytotoxic T-lymphocyte antigen 4 (CTLA4), lymphocyte-activation gene 3 (LAG3), T-cell immunoglobulin and mucin-domain containing-3 (TIM3) as well as stimulators such as inducible T-cell CO-stimulator (ICOS)^2,3^. These receptors and ligands play crucial roles in regulating immune cell functions and maintaining immune homeostasis^4^. However, in cancer, these checkpoints are often overactivated, leading to impaired antitumor immunity^5^. Inhibiting the activation of ICPs to restore immune cell function has been utilized in the clinical treatment of cancer; for example, in 2011, the Food and Drug Administration (FDA) authorized ipilimumab for the treatment of advanced unresectable melanoma^6,7^. Nonetheless, researchers acknowledge current issues with immune checkpoint-related therapies, such as variability in patient benefits, resistance in some patients, and the nonnegligible incidence of severe autoimmune symptoms^8,9^. Therefore, the exploration of safer and more effective ICB therapies is urgently needed. This requires a deeper understanding of the mechanisms of action of ICPs.

Current studies posit that ICPs contribute to tumor immune evasion via the downstream signals they mediate. The processes of their expression, transport, activation, and degradation are influenced by a plethora of ‘messengers’ within the tumor microenvironment (TME)^3,10,11^. EVs are phospholipid membrane-coated particles that selectively carry cytoplasmic components, such as proteins, RNA, and lipids, derived from donor cells, and EVs are considered potentially the most complex form of intercellular communication^12^. According to the Minimal Information for Studies of Extracellular Vesicles (MISEV) guidelines that were introduced by the International Society for Extracellular Vesicles (ISEV) in 2014 and updated in 2018, EVs can be classified from multiple dimensions. Based on their biogenesis, EVs can be classified as exosomes derived from endosomes or microvesicles originating from the plasma membrane^13^. EVs are believed to play significant roles in promoting tumor cell proliferation, angiogenesis, metastasis, immune suppression, and drug tolerance^14,15^.

In particular, the cargoes that are carried by EVs have been found to exert profound regulatory effects on the activation, proliferation, secretion, and apoptosis of various immune cells^11,16–18^. Moreover, extensive researches have shown that these functions can be achieved by affecting ICPs on target cells (Table 1). ICP-related EVs also demonstrate immense potential in tumor therapy; for example, EVs carrying ICPs can be used to monitor the response to ICB therapy and the prognosis of patients with tumors^19–21^, and certain cargoes within EVs have been found to be capable of downregulating ICPs on immune cell surfaces, thereby facilitating tumor immunity or enhancing ICB therapeutic efficacy^22–24^. Given the pivotal roles of EVs in the spectrum of ICP functions, in order to increase the efficacy and safety of ICB therapies, further exploration of how EVs influence ICPs is crucial. In this review, we comprehensively describe the interactions between EVs and major ICPs, elucidating how EVs influence the production and transportation of ICPs, as well as the roles of ICPs that are carried by EVs. Additionally, we review the efforts made to apply ICP-related EVs in clinical practice. Our aim is to shed light on potential directions for future research on the link between EVs and ICPs, offering insights for advancing tumor therapy.

**Table 1:** EVs regulate the biogenesis of immune checkpoints.

Type of cancer	Origin	Cargos	Target Cells	Target Immune Checkpoints	Mechanism	impacts on cancer progression	References
Hepatocellular Carcinoma	Cancer cell derived EVs	LOXL4	M2-polarized tumor-associated macrophages	PD-L1	Induce PD-L1 expression by activating STAT1	Promoted the immune escape of HCC cells by inhibiting the killing ability of CD8 + T cells	^197^
	Cancer cell derived EVs	GOLM1	cancer cells	PD-L1	Promote CSN5-mediated PD-L1 deubiquitination	Promotes PD-L1 stabilization	^60^
			macrophages/NK cells		Upregulate PD-L1 expressed by TAMs	Suppresses NK cells	
			cancer cells		Suppresses Rab27b diverts PD-L1 to the membrane	Increase the exosomal PD-L1 levels	
	M1-polarized tumor-associated macrophages derived EVs	MISP	cancer cells	PD-L1	Upregulated phosphorylation of STAT3	Advance immune escape of HCC cells	^30^
Ovarian Cancer	M2-polarized tumor-associated macrophages derived EVs	NEAT1	cancer cells	PD-L1	Sponged miR-101-3p to increase translate factor ZEB1 and PD-L1 expression	Promote OC cell proliferation and CD8 + T cell apoptosis	^34^
Esophageal Squamous Cell Carcinoma	Cancer cell derived EVs	PIK3CB	cancer cells	PD-L1	Promoted the transcriptional activity of the PD-L1 promoter	Promote epithelial-mesenchymal transition	^29^
	Esophageal carcinoma stem cells derived EVs	OGT	CD8 + T cells	PD-1	unknown	Affect the expansion and function of cytotoxic CD8 + T cells	^44^
Breast Cancer	Cancer cell derived EVs	miR-106b-5p	M2-polarized tumor-associated macrophages	PD-L1	Promote phosphorylation of PI3K downstream products by inhibiting PTEN translation	Regulate PD-L1 expression in macrophages and their polarization	^30^
		miR-18a-5p			Promote the binding of STAT3 and PD-L1 coding gene by inhibiting PIAS3 translation		
Head and Neck Cancer	Cancer cell derived EVs	IFNGR1	fibroblastic reticular cells	PD-L1	Activate downstream JAK and STAT1 to promote PD-L1 transcription	Facilitate pre-metastatic niche formation and tumor metastasis in sentinel lymph node	^32^
	Cancer cell derived EVs	miR-21-5p	cancer cells	LAG3	Inhibit expression of LAG3 transcriptional regulatory factor early growth response protin 3	Participate in tumor cell drug resistance and regulate epithelial-mesenchymal transition	^198^
Colorectal Cancer	Colorectal cancer stem cells-derived exosomes	miR-17-5p	cancer cells	PD-L1	Targeted inhibition of SPOP expression, limiting the binding of ubiquitin-activating enzyme to PD-L1	Promote growth and inhibit anti-tumor immunity in CRC cells through promoting PD-L1	^51^
	Cancer cell derived EVs	LncRNAKcnq1OT1		PD-L1	LncRNAKcnq1OT1 competitively binds with MIR-30A-5P to Ubiquitin-Specific Protease 22 (USP22), thereby facilitating the cleavage of ubiquitin chains from ubiquitinated PD-L1 by USP22	Suppress basic apoptosis rate of tumor cells	^53^
	Cancer cell derived EVs	miR-21-5p	M2-polarized tumor-associated macrophages	PD-L1	Promote phosphorylation of PI3K downstream products by inhibiting PTEN translation	Regulate PD-L1 expression in macrophages and their polarization	^199^
	Cancer cell derived EVs	miR‐200a		PD-L1	Promote phosphorylation of PI3K downstream products by inhibiting PTEN translation; Promote phosphorylation of STAT1 by inhibiting SOCS1 translation		
Lymphoma	Cancer cell derived EVs	unknown	CD8 + T cells	CTLA4	Promote CTLA4 expression with unknown mechanism	Transform effect CD8 + T cells into regulatory CD8 + T cells with active immunosuppressive function, secreting inhibitory cytokines IL10 and TGF-β	^98^
		unknown	Chimeric antigen receptor modified CD8 + T cells	CTLA4	Promote CTLA4 expression with unknown mechanism	Damage tumor killing ability of chimeric antigen receptor modified CD8 + T cells	^99^
Hepatocellular Carcinoma	Hepatic stellate cell derived EVs	circWDR25	hepatic stellate cell	CTLA4	Up-regulates ALOX15 via sponging mir-4474-3p, thereby promote CTLA4 expression	Facilitated HCC cell proliferation and invasion	^103^
Triple-negative Breast Cancer	Cancer cell derived EVs	CCL5	M2-polarized tumor-associated macrophages	CTLA4	Promote CTLA4 expression with unknown mechanism	Remodel the surrounding tumor stroma and immune infiltrate; increase tumor metastasis to the lung	^200^
Nasopharyngeal Carcinoma	Cancer cell derived EVs	Galectin-9	Dendritic cells	TIM3	Binding to TIM3 on the surface of dendritic cells induces the generation of regulatory dendritic cells.	Regulatory dendritic cells with mature phenotype inhibit T cell proliferation and secrete high level of IL-4.	^112^
Advanced Lung Cancer	Malignant pleural effusion derived EVs	CEACAM1	CD3 + CD4-CD8-double-negative T cells	TIM3	Binding to TIM3 on the surface of double-negative T cells downregulates their ability to kill tumors.	Create immunosuppressive environment for intrathoracic metastasis of tumors.	^115^
Epithelial Ovarian Cancer	Tumor-associated macrophages derived PD-L1+ exosomes	PD-L1	CD8 + T cells	LAG3	Unknow, possibly from the increased expression of CPT1A in CD8 + T cells that lead to an increase in reactive oxygen species-related cellular damage	Promote the apoptosis of CD8 + T cells	^87^
Lung Adenocarcinoma	Adipocyte‐derived exosomes	miR‐27a‐3p	CD4 + T cells	ICOS	Inhibit the transcription of ICOS	Reduce the release of IFN-γ by CD4 + T cells.	^134^

## Crosstalk between EVs and PD-L1

**Fig. 1:**
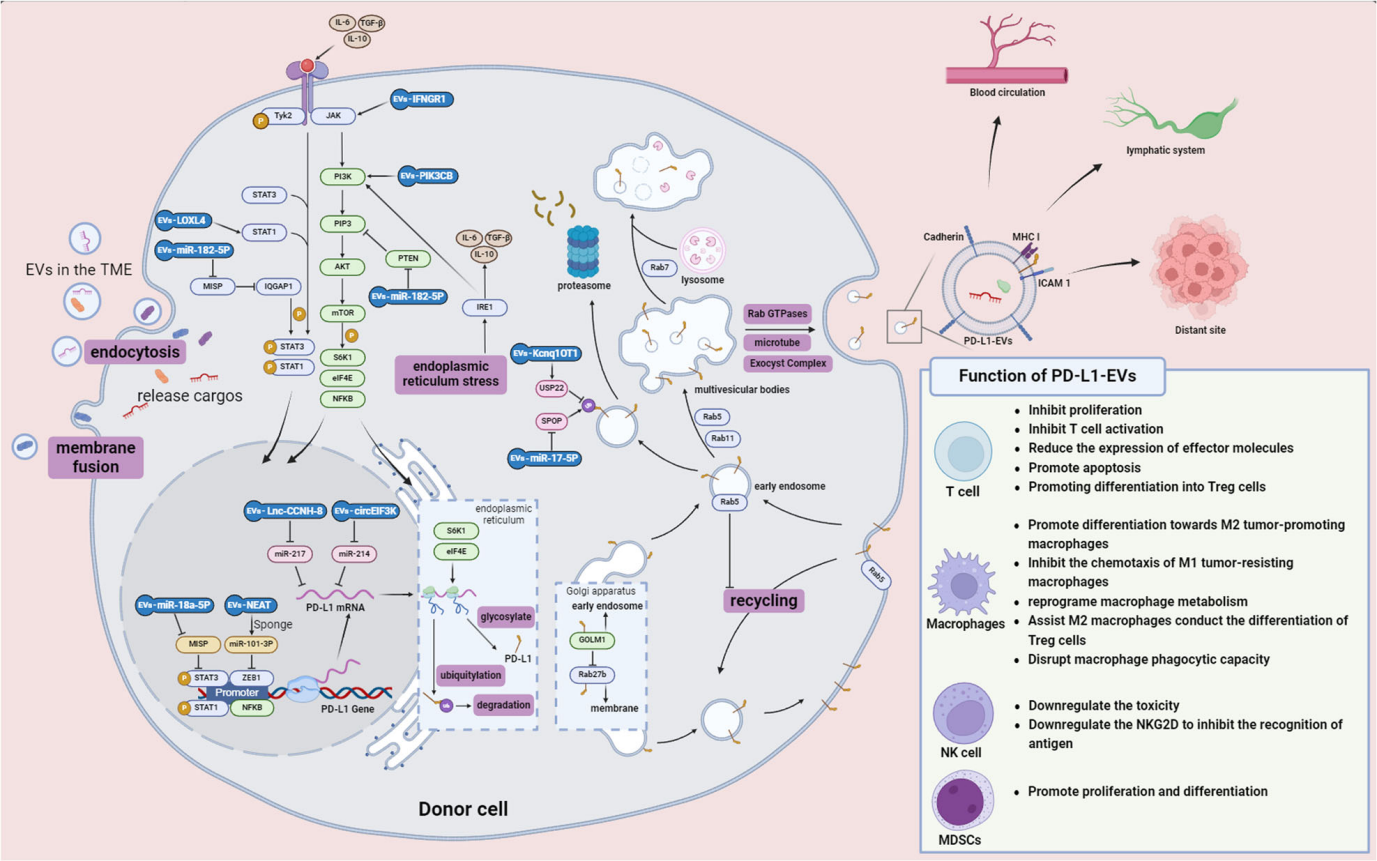
Dynamic Role of PD-L1-Expressing Extracellular Vesicles (EVs) in the Tumor Microenvironment and Their Impact on Immune Cells. EVs can enter recipient cells through endocytosis or membrane fusion, releasing their cargo to influence multiple pathways associated with PD-L1 expression. The transcription of PD-L1 is regulated by various signaling and endoplasmic reticulum stress pathways. In the nucleus, PD-L1 mRNA is also regulated by multiple noncoding RNAs that are carried into the cell by EVs. Once inside the cytoplasm, cells selectively sort PD-L1 into extracellular vesicles. These PD-L1-expressing EVs eventually diffuse into the blood, lymph nodes, and even distant metastatic sites, affecting the functions of various immune cells. For example, PD-L1 EVs can inhibit T-cell activation, promote Treg cell differentiation, suppress effector molecule expression, and induce apoptosis to inhibit T-cell proliferation. In macrophages, PD-L1 EVs can promote macrophage differentiation toward the M2 tumor-promoting phenotype, inhibit the chemotaxis of M1 tumor-resistant macrophages, reprogram macrophage metabolism, and disrupt the phagocytic capacity of macrophages. For NK cells, PD-L1 EVs can reduce toxicity and inhibit NKG2D antigen recognition. Additionally, they can promote the proliferation and differentiation of myeloid-derived suppressor cells (MDSCs).

PD-L1 (B7-H1 or CD274) is a 290-amino acid protein that belongs to the type I transmembrane protein receptor B7 family. It is expressed on the surfaces of various cell types, including antigen-presenting cells (APCs), T cells, B cells, monocytes, and epithelial cells^25,26^. When PD-L1 specifically binds to PD-1 on the surface of target cells, it inhibits downstream pathways related to immune cell activation and proliferation, such as the PI3K/AKT/mTOR and RAS/MEK/ERK1/2 pathways, by recruiting Src homology region 2-containing protein tyrosine phosphatase 2 (SHP2)^3^. Extensive researches have demonstrated a close association between PD-L1 and EVs; EVs can increase the expression of PD-L1 and facilitate its intercellular transport, and in turn, PD-L1 is selectively packaged into EVs to exert its effects (Fig. 1).

### EVs Regulate PD-L1 Biogenesis

Under the regulation of transcription factors and cis-acting elements, PD-L1 is expressed and then transported to the surface of the endoplasmic reticulum. After translation and posttranslational modification, PD-L1 is delivered to the cell surface^27^. A significant portion of PD-L1 is subsequently loaded into EVs and released from the cell. The specific mechanisms are detailed in Fig. 1. The regulators of PD-L1 transcription that are carried by EVs are diverse, and current studies have focused mainly on PI3K/Akt pathway components and signal transducer and activator of transcription 1/3 (STAT1/3) proteins. The PI3K/Akt pathway, which is commonly hyperactivated in tumor cells, plays critical roles in cancer cell proliferation, immune evasion, and drug resistance. It primarily activates PD-L1 transcription by phosphorylating the downstream transcription factor NFκB^28^. EVs from esophageal squamous cell carcinoma cells carrying the PI3K catalytic subunit PIK3CB enters other tumor cells, promoting PD-L1 promoter transcriptional activity^29^. STAT1/3, which are downstream transcription factors of interferon I, are also involved in EV regulation. EVs derived from various tumor cells carry protein or RNA cargo that promotes PD-L1 expression by increasing the phosphorylation of STAT1/3 or by assisting these transcriptional regulators in binding to the PD-L1 gene^30,31^. Additionally, under normal conditions, fibroblastic reticular cells directly interact with T cells in lymph nodes, providing survival signals. EVs from head and neck squamous cell carcinoma cells transport phosphorylated interferon gamma receptor 1 (IFNGR1) to fibroblastic reticular cells within sentinel lymph nodes, activating the JAK/STAT1 pathway without binding to IFNG, thereby promoting PD-L1 transcription^32^. The functional transformation of these EVs in the tumor microenvironment indicates that the cargo that is carried by EVs can subvert the body’s antitumor immune response and create a favorable survival environment that facilitates tumor lymph node metastasis via PD-L1^33^.

The regulation of PD-L1 transcription is not limited to these pathways. For example, EVs that are derived from M2 macrophages carry the long noncoding RNA NEAT1 sponge miR-101-3p, which upregulates the transcription factor zinc finger E-box-binding homeobox 1 (ZEB1), thereby promoting PD-L1 expression by tumor cells^34,35^. EV cargo can also target microRNAs that directly act on PD-L1 mRNA. For example, EVs from hypoxic tumor-associated fibroblasts carry circEIF3K, which can enter tumor cells and downregulate the ability of miR-214 to target PD-L1^36^. EVs from hepatocellular carcinoma (HCC) cells carry Lnc-CCNH-8, which acts as a sponge for miR-217^37^. The unstable intracellular environment caused by active protein synthesis in tumor cells, as well as the production of abundant reactive oxygen species (ROS) due to radiotherapy and chemotherapy, can lead to protein folding abnormalities in the endoplasmic reticulum (ER), resulting in endoplasmic reticulum stress (ERS)^38^. Although excessive stress can induce apoptosis in tumor cells, in most cases, tumor cells restore intracellular homeostasis by increasing ER efficiency and degrading abnormally folded proteins, thereby increasing survival^39^. EVs from head and neck squamous cell carcinoma cells undergoing ERS induce macrophage M2 polarization by upregulating the PI3K/AKT pathway^40^. Subsequently, increased levels of IL-6 and IL-10 synergistically activate the ERS-related IRE1α–XBP1 signaling pathway, which further contributes to the expression of inflammatory factors and promotes the expression of PD-L1 in M2 macrophages^41^. This finding was validated in another study^42^; EVs derived from breast cancer cells undergoing ERS carry miR-27a-3p, which targets the ERS-related protein MAGI2 to downregulate PTEN, thereby promoting PD-L1 expression in M2 macrophages. Interestingly, upon receiving endoplasmic reticulum stress signals, M2 macrophages experience lysosomal overload, leading to the potential extracellular release of numerous proteases, including cathepsins. This can undermine the stroma’s ability to restrict the tumor^42^. Concurrently, this reduces the adhesion of EVs to the stroma, potentially allowing more EVs to diffuse to prospective metastatic sites and assisting in tumor metastasis. The hypoxic tumor microenvironment prompts an increase in ROS, which can modify EV-mediated cellular communication, thereby modulating PD-L1 expression. For example, the accumulation of ROS in the peritoneal metastases of ovarian cancer prevents M2 macrophages from absorbing tumor-derived EVs carrying miR-155-5p, preventing the downregulation of PD-L1^43^. Unfortunately, research on the role of EVs in the regulation of PD-1 transcription is limited. Yuan et al.^44^ reported that EVs from esophageal squamous cell carcinoma stem cells carrying the nutrient sensor O-GlcNAc transferase (OGT) enter CD8^+^ T cells to promote PD-1 expression, indicating that EVs are indeed involved in the transcription of PD-1, which may be a future research direction.

Posttranscriptional regulation and posttranslational modification of PD-L1 are quality control measures for its intracellular transport and are considered crucial mechanisms for tumor immune suppression^45^. Notably, the N-glycosylation of PD-L1 appears to be of paramount importance. Studies have indicated that N-glycosylation of PD-L1 is directly related to its expression level on the cell surface, with glycosylation preventing the proteasomal degradation of PD-L1 after its interaction with glycogen synthase kinase 3β (GSK3β)^46–48^. Zhu et al.^49,50^ reported that the glycosylation of PD-L1 on the surface of EVs is a prerequisite for its binding and interaction with PD-1, making glycosylated PD-L1 on patient EVs a more reliable potential tumor biomarker. The ubiquitination of PD-L1 mediates its proteasome-dependent degradation, and EVs in the tumor microenvironment also inhibit this process, thereby increasing PD-L1 expression levels. Speckle-type POZ protein (SPOP) is one of the primary E3 ubiquitin ligases that mediates PD-L1 ubiquitination degradation, transferring E2 ubiquitin-conjugating enzymes and E1 ubiquitin-activating enzymes to PD-L1. Colorectal cancer stem cell-derived EVs carry miR-17-5p to inhibit SPOP expression, thereby inhibiting intracellular PD-L1 ubiquitination-mediated degradation^51,52^. Conversely, tumor cell-derived EVs carry the long noncoding RNA Kcnq1OT1, which competes with miR-30A-5p to bind to ubiquitin-specific protease 22 (USP22), increasing USP22 expression and severing the link between PD-L1 and ubiquitin chains^53^. However, many questions remain regarding the role of EVs in the ubiquitination of PD-L1, and there is a lack of research on whether EVs also participate in the ubiquitination-mediated degradation of PD-1.

### EVs Carry PD-L1 into the Tumor Microenvironment

PD-L1, which is a membrane-bound ligand, has been extensively studied and found to rely primarily on EVs for its intercellular transport^54–56^. PD-L1 on the plasma membrane undergoes continuous endocytosis. Some internalized PD-L1 proteins return to the cell surface, whereas others are loaded into EVs along with endoplasmic reticulum proteins^12,57^. The strong colocalization of PD-L1 with the EV marker CD63 within multivesicular body (MVB) substructures suggests that donor cells selectively load PD-L1 into EVs^58^. The sorting of PD-L1 to the plasma membrane or early endosomes begins in the Golgi apparatus, where overexpressed Golgi membrane protein 1 (GOLM1) binds directly to PD-L1 for transport. Ras-associated proteins (Rabs) are part of the Ras superfamily of small GTPases and play an important role in producing secretory forms of molecules within cells^59^; among them, Rab27b facilitates the transport of PD-L1 to the cell surface, whereas GOLM1 downregulates Rab27b levels, restraining PD-L1 within the trans-Golgi network area and ultimately leading to its entry into early endosomes^60^. The membranes of early endosomes invaginate to form MVBs, creating intraluminal vesicles (ILVs) that carry PD-L1. By increasing the efficiency of this process, donor cells can facilitate the release of PD-L1-EVs. For example, Rab5 colocalizes with internalized PD-L1 on the cell membrane, aiding in its transport to MVBs and inhibiting its retransport back to the cell surface^61^. The downregulation of histone lysine-specific demethylase 1 (LSD1) in gastric cancer cells reduces the number of MVBs and the secretion of EVs containing PD-L1, as does the downregulation of Rab11, which facilitates MVB maturation^62,63^. Surprisingly, this also decreases the intracellular expression of TSG101, a component of the endosomal sorting complex required for transport, which binds to the surface of MVBs to induce their degradation through lysosomes^57,64^; these findings suggest complex and but unknown regulatory mechanisms underlying the assembly of PD-L1-containing EVs. ERS in donor cells can also promote the formation of MVBs. The expression levels of the ERS-related proteins inositol-requiring enzyme 1 (IRE1) and PKR-like ER kinase (PERK) are increased in tumor cells, promoting the phosphorylation of proteins related to MVB formation^65^.

Mature MVBs carrying PD-L1 can fuse with lysosomes for degradation or be transported to the cell surface for release^66^. Fusion with lysosomes is dependent on Rab7, whereas transport to the cell surface has recently been linked to the exocyst complex, an octameric protein complex that binds to phosphatidylinositol 4-phosphate (PI4P) and Rab11 on MVB surfaces. The Exo84 subunit of the exocyst complex then assembles with the Sec3 subunit on the inner side of the plasma membrane, anchoring MVBs for release^67^. Additionally, the Exo70 subunit within this complex is also directly involved in the transport of MVBs to the plasma membrane^68^. P53, the central regulator of the DNA damage response in tumor cells, is another factor that is closely associated with MVB release in response to environmental stress in tumor cells. P53 regulates the fusion of MVBs with the plasma membrane through the transmembrane protein TASP6, thereby increasing the number of EVs loaded with PD-L1^69,70^. However, the high expression of cytotoxin-associated gene A (CagA) in *Helicobacter pylori*-related gastric cancer cells has been found to increase PD-L1 content in EVs by downregulating P53, suggesting additional regulatory functions of P53 in the content of PD-L1 in EVs^71^. The opening of calcium channels, specifically QRAI1, on the surface of lung cancer cells provides a stable calcium environment for the function of Rab27a and synaptotagmin-like protein 2 (SLP2-a) during the fusion of MVBs containing PD-L1 with the plasma membrane^55^. In summary, tumor cells create an optimal environment for selectively packaging PD-L1 into EVs, thereby facilitating its function. Notably, PD-1 is also internalized from the cell membrane, but very few studies have attempted to explore whether it can be repackaged into EVs or to determine the regulatory role of donor cells in this process^5,72^.

EVs in the tumor microenvironment, despite lacking active motility, can accumulate at specific sites under the influence of adhesion-related proteins^12^. EVs carrying PD-L1 express fewer adhesion proteins that bind to fibronectin and collagen in the extracellular matrix, which reduces their retention by the tumor extracellular matrix and allows rapid dispersion beyond the tumor tissue^73^. Conversely, these EVs express high levels of cadherins, which are thought to mediate adhesion to various endothelial cells, ultimately guiding EVs to enter lymph nodes, the vascular system, and distant metastatic sites rich in endothelial cells, such as lung tissue^73–75^. EVs carrying PD-L1 also express high levels of the intercellular adhesion molecule 1 (ICAM1) and MHC class I molecules, which, along with PD-L1, form immune synapses to specifically bind to CD8^+^ T cells, thereby attacking T cells within lymph nodes^73,76^. The unique biological behavior exhibited by PD-L1-expressing EVs may represent a potential direction for future targeted therapeutic approaches.

### PD-L1 Exerts Functions via EVs

PD-1/PD-L1 signaling plays a critical role in shaping the immunosuppressive tumor microenvironment^3,5^. However, the efficiency of signal transduction activated by cell surface PD-L1/PD-1 interactions is limited by the cell contact surface area. Consequently, cells within the tumor microenvironment cleverly load PD-L1 onto EVs. This strategy not only provides more available PD-L1 binding sites on the target cell surface but also allows PD-L1 to exert its effects beyond the location of the donor cell within the microenvironment^73^. Numerous studies have demonstrated that PD-L1 on EVs can influence the function of immune cells (Fig. 1)^54,77,78^. The immunosuppressive mechanisms of PD-L1-EVs appear to mirror those of membrane-bound PD-L1. They primarily inhibit CD8^+^ T-cell proliferation, downregulate the production and release of effector molecules, and promote T-cell apoptosis^79^. They also inhibit the proliferation of CD4^+^ T cells while promoting their differentiation into regulatory T cells^80,81^. The main effect of tumor-associated macrophages is to promote their differentiation toward the M2 tumor-promoting phenotype^82^. Furthermore, PD-L1-EVs from head and neck squamous cell carcinoma cells suppress B-cell proliferation and activity and increase PD-1 and LAG3 expression on their surface; PD-L1-EVs from gastric cancer cells promote myeloid-derived suppressor cell expansion and differentiation, suggesting that a wide range of immune cells can be affected by PD-L1-EVs^83,84^. Additionally, PD-L1-EVs perform unique functions. The diffusive nature of EVs in the TME allows PD-L1 to be not only expressed by one cell but also transferred to other cells. Mauro et al. reported that administering exogenous PD-L1-EVs could rescue the growth of tumors that are incapable of independently secreting exosomes. Moreover, in prostate cancer, tumors with low PD-L1 expression actively take up PD-L1-EVs from high PD-L1-expressing tumor cells, thereby enhancing overall antitumor immunity^85,86^. PD-L1-EVs from head and neck squamous cell carcinoma establish a positive feedback loop between M2 macrophages and Treg cells in the TME, further suppressing CD8^+^ T-cell proliferation^56^. PD-L1-EVs also contribute to the establishment of metastatic niches; for example, M2 macrophages in ovarian cancer appear in the peritoneal cavity even before tumor cells arrive, where they target carnitine palmitoyl-transferase 1 A expression in T cells within the peritoneal cavity through the PD-L1-EVs they release. This enhances fatty acid oxidation in T cells, increases the levels of intracellular reactive oxygen species, and subsequently damages T cells^87^.

PD-L1-EVs play a critical role in resistance to ICB therapy. High levels of PD-L1-EVs in patients with cancer suppress the proliferation and activation of T cells, with some T cells exhibiting irreversible functional exhaustion, inherently limiting the effectiveness of anti-PD-L1 treatments^88^. Though T cells rescued by anti-PD-L1 therapy can continue to exert antitumor effects, the IFN-γ that is secreted by activated T cells further promotes the transcription of PD-L1. Additionally, the stimulatory effect of activated T cells on tumor cells prompts the production of more EVs, thereby increasing the levels of PD-L1-EVs and forming a mechanism that counteracts the efficacy of anti-PD-L1 therapy^89^. The ability to release PD-L1-EVs varies across different tumor types and states, potentially contributing to the heterogeneous efficacy of anti-PD-L1 treatments^90^. Interestingly, researchers reported that inhibiting Rab27a to downregulate EV production greatly increased the efficiency of binding between PD-L1 on the tumor cell surface and anti-PD-L1 antibodies. In vivo experiments demonstrated a dose-dependent reduction in the quantity of PD-L1 on the tumor surface that was bound to anti-PD-L1 antibodies by PD-L1-EVs, suggesting that PD-L1 on EVs has greater affinity for anti-PD-L1 antibodies than for PD-L1 on the cell surface^91^. A previous study^92^ revealed that tumor cells can produce abnormally spliced PD-L1 that lacks the transmembrane domain, which is secreted extracellularly and displays high affinity for anti-PD-L1 antibodies, indicating that tumor-derived PD-L1 is not constant and may undergo changes during packing into EVs or at certain biological stages; this finding warrants further research on the increased affinity of PD-L1 for anti-PD-L1 antibodies. Moreover, the specificity of adhesion molecules on PD-L1-EV surfaces, especially the high expression of cadherins and ICAM1 in these EVs and their tendency to disperse into the circulation, suggest that circulating PD-L1-EVs could constitute the first line of defense that weakens the efficacy of anti-PD-L1 antibodies. Indeed, the levels of PD-L1-EVs in the plasma of patients with melanoma were found to be negatively correlated with responsiveness to anti-PD-L1 antibody therapy^93^. Recently, combining ICB with other radiotherapy and chemotherapy modalities has emerged as a new treatment approach, but PD-L1-EVs have also been found to contribute to tolerance to these therapies. For example, head and neck squamous cell carcinoma lines presented an increase in PD-L1-EVs after fractionated radiotherapy or cisplatin treatment, along with a reduction in apoptosis^94^. The number of glioblastoma stem cell-derived PD-L1-EVs increased after treatment with temozolomide, increasing intracellular PD-L1 levels after ingestion by tumor cells and subsequently inducing AMPK/ULK1 pathway-mediated protective autophagy, resulting in tolerance to temozolomide by clearing damaged organelles within cells^95^.

## Crosstalk between EVs and CTLA4

CTLA4 (CD152) is a type I transmembrane glycoprotein that is homologous to the immunoglobulin CD28. Unlike PD-L1, which functions primarily through EVs, CTLA4 exerts its immunosuppressive effects by continuous flipping between the inside and outside of the cell membrane to bind to its ligands. Owing to efficient endocytosis, CTLA4 accumulates in the cytoplasm. Upon the production of costimulatory signals generated by the binding of CD28 with its ligands CD80/CD86 and the TCR with MHC, CTLA4 is transported to the cell surface, where it competes with CD28 to bind to CD80/CD86, thereby mediating T-cell nonresponsive conditions and recruiting SHP2 to activate T-cell inhibitory signals^96^. CTLA4 is predominantly expressed on the surface of regulatory T cells and activated CD8^+^ T cells, and EVs in the tumor microenvironment further upregulate its expression to create an inhibitory milieu^3,97^. EVs derived from lymphomas increase CTLA4 expression on CD8^+^ T cells, converting them into regulatory CD8^+^ T cells that perform immunosuppressive functions and secrete the inhibitory cytokines IL10 and TGF-β^98^. However, it is unclear whether this upregulation occurs through increased transcription and translation of CTLA4 or by promoting its presentation on the cell surface. EVs carrying lymphoma surface antigens can preferentially bind to chimeric antigen receptor-modified T cells that are used in therapy, increasing CTLA4 expression on their surface and impairing their tumor-killing ability^99^. It appears that ICPs can also interfere with each other through EVs. PD-L1-EVs from esophageal squamous cell carcinoma decrease the ratio of circulating follicular helper T cells to follicular regulatory T cells and upregulate CTLA4 in the former, inhibiting follicular helper T cell differentiation by preventing the binding of CD28 to its ligands, leading to high expression of ICOS and activation of downstream PI3K^100,101^. As more studies have shown that CTLA4 is distributed on other cell surfaces, the related roles of EVs are expanding^102^ and EVs derived from hepatic stellate cells carrying circWDR25 enter other stellate cells, sponge miR-4474-3p, and upregulate ALOX15, thereby promoting CTLA4 expression^103^.

In recent years, studies have shown that CTLA-4 is loaded into EVs, where it plays a role in suppressing T-cell function and promoting T-cell apoptosis^104,105^. EVs derived from triple-negative breast cancer cells carry CTLA-4, which can induce irreversible apoptosis in CD8^+^ T cells. However, the use of anti-CTLA-4 antibodies does not significantly mitigate this effect, suggesting that the apoptotic effect of CTLA-4-EVs is likely mediated by the stress-induced apoptosis of T cells triggered by the influx of extracellular components, including CTLA-4^104^. Furthermore, EVs from tumor cells of patients with cachexia and hepatocellular carcinoma carrying CTLA-4 have been shown to promote tumor cell proliferation and metastasis through the PTEN/CD44 pathway within the tumor microenvironment^106^. These findings indicate that the functionality of this membrane-bound receptor is further expanded upon its incorporation into EVs.

## Crosstalk between EVs and TIM3

TIM3 is a member of the T-cell immunoglobulin and mucin domain (TIM) gene family and is classified as a type I transmembrane protein. It is composed of an N-terminal immunoglobulin variable (V) domain and five atypical cysteines, a mucin stalk, a transmembrane domain, and a cytoplasmic tail^3,107^. Its immunosuppressive function relies on its interactions with multiple ligands, including C-type lectin galectin-9 (Gal-9), high mobility group box 1 (HMGB1), carcinoembryonic antigen-related cell adhesion molecule 1 (CEACAM1), and the nonprotein ligand phosphatidylserine (PS)^108^. Each of these ligands has a specific mechanism for binding to TIM3, leading primarily to the phosphorylation of its cysteine residues. Following ligand binding, HLA-B-associated transcript 3 (BAT3) disassociates from the cytoplasmic tail, allowing the tyrosine kinase FYN to bind and generate T-cell inhibitory signals. However, extensive researches have confirmed that different ligands can induce unique effects upon binding to TIM3 under various conditions^3,109^. TIM3 is expressed on a variety of cell surfaces and is found predominantly on T cells, NK cells, macrophages, dendritic cells, and mast cells^107^. It is highly expressed on NK cells but functions mainly in T-cell suppression, is localized on T-cell membrane rafts and is recruited to immunological synapses upon T-cell activation to exert its inhibitory effect^110,111^. Recent studies have shifted this perspective, showing that TIM3 expression on various myeloid-derived cells appears to be greater than that on T cells^112^, contributing to a systemic immunosuppressive environment through mechanisms such as promoting M2 macrophage differentiation and inhibiting DNA recognition receptors in antigen-presenting cells^113,114^.

The interaction between TIM3 and EVs is notably complex, with several ligands found to be transported via EVs. EVs derived from nasopharyngeal carcinoma cells carrying Gal-9 bind to TIM3 on dendritic cells (DCs), transforming them into regulatory DCs. This interaction downregulates the release of costimulatory molecules and inflammatory factors while concurrently increasing the expression of PD-1 and CTLA4 on their surface. Additionally, Gal-9-carrying EVs inhibit the migration of DCs, thereby impairing their ability to efficiently enter the tumor microenvironment and exert their functions^112^. A recent study has suggested that the binding of Gal-9 to TIM3 on the surface of DCs does not directly activate downstream inhibitory signals. Instead, its dimerization domain promotes the aggregation of TIM3 on the DC surface, enhancing the binding efficiency of the HMGB1-antigen DNA complex to TIM3. This, in turn, competitively inhibits the binding of HMGB1 to endocytic receptors, thereby preventing the entry of tumorigenic DNA into DCs and the induction of the antigen-presenting response^113^. However, research on this EV-mediated mechanism is still lacking. Given the long-distance, multisite transport capabilities of EVs, TIM3-related EVs could regulate tumor immune evasion beyond the TME^115^. In pleural effusions from patients with lung cancer, EVs not only express prometastatic angiogenic proteins but also exhibit high surface expression of CEACAM1. These EVs bind to TIM3 on the surface of CD3^+^CD4^-^CD8^-^ double-negative T (DNT) cells in the effusion, diminishing their cytotoxic effect on free tumor cells within the malignant effusion and thus fostering a conducive environment for tumor metastasis^115,116^. TIM3 itself can also be transferred between cells via EVs, a phenomenon observed in osteosarcoma and melanoma. TIM3-EVs suppress the function of CD4^+^ T cells and induce the differentiation of M2-type macrophages. Unlike CTLA4-EVs, EVs carrying TIM3 seem to perform functions similar to those of its membrane-bound form^117,118^. Although the functional mechanisms of TIM3-EVs remain unclear, it is hypothesized that they primarily increase the expression of TIM3 within target cells after entry, but this requires further experimental validation. In summary, considering the numerous ligands and complex modes of action associated with TIM3, its exploration presents a compelling area of research.

## Crosstalk between EVs and LAG3

LAG3 (CD223) plays a pivotal role in limiting T-cell activation, and inhibitors that target LAG3 represent the third ICB therapeutic protocol approved by the FDA. When used in combination with anti-PD-1 antibodies for the treatment of patients with melanoma, these agents have demonstrated substantial efficacy and safety, with significant clinical potential^119^. LAG3, an inhibitory receptor belonging to the type 1 Ig superfamily, has a structure similar to that of CD4 but has a greater affinity for MHC class II molecules^108^. Upon antigen activation of the TCR/CD3 complex, binding with LAG3 is mediated through a KIEELE motif, a glutamic acid‒proline dipeptide repeat (EP motif), and a serine phosphorylation site (S484) in the cytoplasmic domain of LAG3, which mediates downstream T-cell inhibitory signals or inhibits TCR signal transduction by disrupting the association of the CD4/CD8 coreceptor with cytoplasmic tyrosine kinases^3,120^. Research on the interaction between LAG3 and EVs is limited, but a substantial body of evidence confirms that immunosuppressive EVs can increase the expression of LAG3 on T-cell surfaces, indicating that this effect is dependent on the miRNAs carried by EVs, which increase LAG3 transcription^87,121^. Posttranscriptional regulation of LAG3 has been found to be associated with N6-methyladenosine (m6A), particularly through the balance between the RNA-binding enzyme YTHDF1, which recognizes m6A sites on LAG3 mRNA and assists in translation, and the RNA demethylase ALKBH5^122^. A recent study on M2 macrophage-derived EVs has indicated that they carry miR-21-5p, which inhibits the expression of the m6A methyltransferase METTL3, thereby limiting mRNA methylation. Although direct evidence of the influence of METTL3 on LAG3 expression is lacking, the regulatory effect of EVs on m6A could also impact LAG3 expression^123^. Tumor endothelial cell-derived EVs that enter circulation can induce high expression of the exhaustion marker LAG3 on T cells at distant sites, creating an immunosuppressive environment that is favorable for tumor metastasis; this shows how EVs can assist in establishing systemic immunosuppression associated with LAG3^97^.

Other ligands for LAG3 have been identified, including galectin-3 (Gal-3), liver and lymph node sinusoidal endothelial cell c-type lectin (LSECtin), fibrinogen-like protein 1 (FGL1), and preformed fibrils of α-synuclein (α-syn PFF); however, the roles of these ligands within the functional spectrum of LAG3 remain controversial^120^. For example, FGL1 has been found to interact specifically and physiologically with LAG3 with high affinity and can mediate LAG3’s T-cell inhibitory function independently of MHC class II, although this conclusion is disputed across different studies^124,125^. However, FGL1 levels are widely believed to be associated with poor patient prognosis, especially as recent study has shown that FGL1 levels in circulating EVs from patients with lung adenocarcinoma more sensitively reflect tumor progression and correlate with responsiveness to anti-PD-1 therapy than free FGL1 in plasma^126^, suggesting a complementary immunosuppressive function of EV-derived FGL1 to PD-L1. Additionally, an increasing number of studies on the role of Gal-3 in mediating T-cell functional suppression via LAG3 have been published^127,128^, with substantial amounts of Gal-3-expressing EVs being extracted from head and neck squamous cell carcinoma cell lines, indicating that Gal-3 may interact with LAG3 through EVs^129^.

## Crosstalk between EVs and ICOS

ICOS is an immune checkpoint expressed specifically on the surfaces of activated CD4^+^ and CD8^+^ T cells. Owing to its structural similarity to CD28 and CTLA4, as well as its ligand B7-H2, which belongs to the B7 protein family alongside PD-L1, ICOS is also referred to as an alternative immune checkpoint^130^. ICB drugs targeting ICOS are in development, but most results indicate that their efficacy is primarily synergistic with those of other ICB medications^131,132^. ICOS mediates tumor progression through T-cell regulation, but this regulatory effect is considered bidirectional; on the one hand, ICOS on Treg cells induces the production of IL-10, mediating immune suppression; on the other hand, ICOS on CD4^+^ and CD8^+^ T cells mediates the production of effector molecules such as IFN-γ and TNFα^133^. Interestingly, adipocyte-derived EVs from obese patients with lung adenocarcinoma carrying miR-27a-3p can directly inhibit ICOS transcription, subsequently reducing the level of IFN-γ released by T cells^134^. Moreover, when circulating Tfh cells and regulatory T (Tfr) cells are cocultured with PD-L1-EVs from esophageal squamous cell carcinoma, ICOS expression increases only on Tfr cells, consistent with the distinct functions of ICOS on these cells^101^. ICOS engagement on Tfh cells can mediate B-cell immune-promoting functions, whereas ICOS engagement on Tfr cells, which is dependent on ICOS for differentiation, inhibits normal B-cell functions upon binding, highlighting the complex nature of ICOS function owing to its opposite effects on different cell types^135,136^. Additionally, ICOS can move between cells via EVs, with EVs released from CD4^+^ T cells selectively enriched with ICOS and CD40 on their surface assisting in the physiological interaction of CD40 with its ligand as an immunostimulant^137^. According to a previous clinical study^138^, patients with persistently high surface expression of ICOS on peripheral blood CD4^+^ T cells who receive CTLA4 blockade therapy exhibited greater clinical benefits. However, a recent study revealed that the levels of ICOS in peripheral blood EVs are unrelated to the response and prognosis of patients with gastric cancer receiving ICB therapy^139^.

## Crosstalk between EVs and other ICPS

The functionality of ICPs is not isolated; in fact, the inhibition of one ICP often leads to compensatory upregulation of other ICPs, posing a significant challenge in current ICB therapy^140^. Exploring novel ICPs is increasingly critical in advancing ICB therapy, and some novel ICPs have been shown to be associated with EVs, suggesting the important role of ICP-related EVs in tumor immunity^141^. B7-H3 (CD276), a member of the B7 family similar to PD-L1 and part of the B7-CD28 interaction, aids in tumor immune evasion and metastasis^142^. Similar to PD-L1, B7-H3 promotes the production of EVs. In medulloblastoma cells, high B7-H3 expression upregulates PIK3C2 (a class II PI3K enzyme), which is related to vesicle formation^143^. In addition, high B7-H3 expression in EVs is correlated with a decreased immune response and adverse patient outcomes^143^. B7-H3 derived from colorectal cancer cell-derived EVs can be taken up by vascular endothelial cells, where it then upregulates and activates the AKT1/mTOR/VEGFA pathway. This process promotes endothelial cell migration and tubule formation, resulting in a different function from that of B7-H3 on the cell surface^144^. Notably, B7-H3 is expressed primarily on antigen-presenting cells, but it is unclear whether EVs from these cells also carry B7-H3 and what their functions are^145^. T-cell immunoreceptor with Ig and immunoreceptor tyrosine-based inhibitory motif (TIGIT) is another kind of coinhibitory receptor that forms an antagonistic network with the costimulatory receptors CD226 (DNAM-1), CD96, and CD112R^146^. As a direct target of Foxp3, TIGIT is considered a Treg marker influenced by immunosuppressive EVs in the tumor microenvironment, indicating active Treg cells^99,147^. Indoleamine 2,3-dioxygenase (IDO), another endogenous immune checkpoint, catalyzes the breakdown of tryptophan to kynurenine (L-kyn), inhibiting effector T-cell activation and promoting Foxp3^+^ Treg differentiation through kynurenine production^148^. IDO can be transferred through EVs, which is correlated with poor outcomes in patients with tumors^149^. In glioblastoma, high IFN-γ expression induces PD-L1 and IDO expression and increases their levels on EVs. However, IDO-EVs do not directly inhibit T-cell function; instead, they induce the differentiation of nonclassical monocytes, thereby indirectly downregulating T-cell proliferation^150^. Furthermore, L-kynurenine can also be loaded into EVs. When these EVs are taken up by endothelial cells, L-kynurenine increases intracellular NAD^+^ levels and Sirt3 acetylation, enhancing mitochondrial function and promoting endothelial cell proliferation, thus inducing tumor angiogenesis^151^. These interactions with EVs expand the functional spectrum of ICPs.

## Clinical prospects of ICP-Related EVs

### EVs as predictors of ICB treatment

Clinical research on ICB therapies has undergone significant advancements in recent years, with the FDA recently approving five drugs that target PD-1 or PD-L1 (nivolumab, pembrolizumab, atezolizumab, durvalumab, and avelumab) for inclusion in treatment guidelines for 11 types of cancer^6^. Drugs that target newly identified ICPs, such as relatlimab and fianlimab that target LAG3, are also being quickly applied in clinic^120,152^. Early-phase clinical trials for ICB drugs that target TIM3 are underway (NCT03680508, NCT05216835). Despite the rapid development of ICB therapies, challenges such as heterogeneous patient responses and common adverse reactions limit the clinical adoption of ICB drugs. Discussions about the safety and efficacy of the earliest FDA-approved CTLA4-blocking drugs, ipilimumab and tremelimumab, continue to this day^153,154^. Although that combination of different ICB drugs has improved therapeutic outcomes, recent reports have highlighted an increased incidence of immune-related adverse events, such as hypophysitis or hypopituitarism^154,155^. Therefore, there is a critical need for biomarkers that can monitor the effects of ICB therapy and predict related adverse reactions. In a pancancer clinical study, researchers reported that PD-L1 levels in tumor tissues can reflect patient responsiveness to ICB therapy^156^, suggesting that ICP expression levels may predict patient benefit from ICB therapy. However, this study relied primarily on genomic analysis and immunohistochemistry of tumor tissues to assess PD-L1 levels. Because most ICPs function as membrane-bound entities and are concentrated in specific locations rather than in circulation, it is difficult to monitor ICP levels via less invasive sample collection methods. EVs, however, serve as an exception; they have the membrane structure that is necessary for ICP attachment and can move within the circulation, offering inherent advantages in monitoring circulating ICP levels. Indeed, liquid biopsies to collect ICP-carrying EVs have been found in multiple studies to increase cancer detection rates and predict patient prognosis^157–159^. Furthermore, researches indicate that the expression of ICPs on the vesicle surface is influenced by disease duration and treatment measures^160–162^. Compared with soluble ICPs, the more stable and accessible carrier form of ICPs on EVs presents significant potential as a biomarker (Table 2).

**Table 2:** EVs as Predictor of immune checkpoint inhibition therapy.

Clinical application	Target immune checkpoint	Cancer involved	Protocol	Sample quantity	Outcome measurement	Reference
Monitoring the efficacy of immune checkpoint blocking therapy	PD-L1	Lung Adenocarcinoma, Lung Squamous Carcinoma, Esophageal Carcinoma, Colorectal Carcinoma, Cholangiocarcinoma, Nasopharyngeal Carcinoma, Lung Small Cell Carcinoma, Lung Large Cell Carcinoma	Combine extracellular vesicles with other serum biomarkers to monitor the therapeutic effect of immune checkpoint therapy	44	Clinical response was determined as best response based on immune-related RECIST (irRECIST). Progression-free survival (PFS) was calculated from the time of treatment till progression or the last follow-up visit.	^201^
	PD-L1	Nonsmall Cell Lung Cancer	Quantification of EVs-PD-L1 levels in patient plasma through the ELISA.	120	The patient’s recurrence-free survival time.	^202^
	PD-L1/PD-1	Nonsmall Cell Lung Cancer	Combine PD-1/PD-L1, NK immune checkpoint markers and cytokines derived from extracellular vesicles to monitor the therapeutic effect of immune checkpoint therapy	17	Tumor response detected on PET-CT 4-6 months after the initiation of treatment.	^149^
	PD-L1	Melanoma	Collecting fecal samples from patients at various time points after the initiation of immunotherapy and measuring the levels of EVs-PD-L1 to assess its relationship with the patients’ treatment response.	20	Incidence and timing of immune-related adverse events; Assessment of immunotherapy response based on irRECIST; Progression-free survival of patients.	^174^
	PD-L1	Nonsmall Cell Lung Cancer	Efficiently and high-purely isolate EVs using Tim4-functionalized magnetic core-shell metal-organic framework (Fe3O4@SiO2-ILI-01@Tim4) that contains a strongly hydrophilic organic ligand 1,3-bis(4-carboxybutyl) imidazolium bromide (ILI). Subsequently, EVs-PD-L1 levels are quantified through high-throughput immunofluorescence assay.	14	The difference in EVs-PD-L1 levels from tumor cell sources between cancer patients and healthy donors.	^19^
	PD-L1	Nonsmall Cell Lung Cancer	By introducing a multi-component nucleotide enzyme linked to a fluorescent reporter protein, a significant amount of fluorescence signal is generated through the activation of the enzymatic cleavage of the fluorescent reporter protein under the simultaneous stimulation of the PD-L1 Aptamer and the Aptamer adhered to EVs’ lipid membrane through hydrophobic lipid affinity.	21	Differences in EVs-PD-L1 levels between patients with progressing tumors and patients without progression following ICB therapy.	^203^
	LAG3	Lung Adenocarcinoma	Indirect monitoring of ICB treatment response by monitoring the levels of the LAG3 ligand FGL1 on plasma EVs	17	Differences in EVs-FGL1 levels among the disease progression group, disease control group, and disease remission group.	^126^
	TIM-3	Hepatocellular Carcinoma	Indirectly monitoring ICB treatment response by monitoring the regulation of TIM3 expression in EVs-circUHRF1.	30	Assess immunotherapy response based on irRECIST and CT scanning.	^179^
As a biomarker for cancer diagnosis	PD-L1	Nonsmall Cell Lung Cancer	First, TiO2 magnetic nanoparticles were combined with exosome phospholipid hydrophilic phosphate heads to capture exosomes indiscriminately. Then the PD-L1 marker “Au@Ag@MBA” required for Surface-Enhanced Raman Scattering (SERS) immunoassay was added to accurately quantify EVs-PD-L1.	29	Differences in EVs-PD-L1 levels among patients with advanced non-small cell lung cancer, early stage cancer, and healthy candidates.	^172^
	PD-L1	Melanoma	developed a dual-target-specific aptamer recognition activated in situ connection system on exosome membrane combined with droplet digital PCR (ddPCR) for quantitation of tumor-derived exosomal PD-L1 (Exo-PD-L1).	45	The difference in EVs-PD-L1 levels from tumor cell sources between cancer patients and healthy donors.	^169^
	PD-L1	Breast Cancer	Two sets of DNA molecular machines are designed to directly amplify the detection signals for ExoPD-L1 protein and ExomiR-21 microRNA in exosome lysates.	16	The relative expression levels of ExomiR-21 and ExoPD-L1 in the plasma exosomes.	^170^
	PD-L1	Nonsmall Cell Lung Cancer	Conjugating the PD-L1 aptamer to ternary metal-metalloid palladium-copper-boron alloy microporous nanospheres capable of conducting electrical signals, to assess the level of EVs-PD-L1 based on its characteristic electrical signal.	10	The difference in EVs-PD-L1 levels from tumor cell sources between cancer patients and healthy donors.	^171^
	LAG3	Lung Adenocarcinoma	Diagnosing lung adenocarcinoma patients by monitoring the levels of LAG3 ligand FGL1 on plasma EVs and assessing its relationship with tumor progression in patients.	69	The differences in EVs-FGL1 levels between healthy individuals and lung adenocarcinoma patients, as well as among patients at different clinical stages.	^126^

Single-molecule array technology has been used for the high-sensitivity detection of protein biomarkers in plasma EVs that are isolated from patients with B lymphoma, and the results revealed that PD-L1-EV levels are correlated with poor patient prognosis and response to chemotherapy, increasing interest in monitoring PD-L1-EVs to assess responses to immune checkpoint therapy^163^. The phenomenon of detecting ICB efficacy through circulating EVs was first identified in melanoma patients treated with pembrolizumab, where plasma PD-L1-EV levels were positively correlated with intracellular Ki-67 levels in CD8^+^ T cells. Moreover, the fold increases in total circulating PD-L1, microvesicle PD-L1, and PD-L1, which are excluded by EVs, were lower in distinguishing responders from PD-L1-EVs^89^.

Traditional EV analysis methods rely primarily on ultracentrifugation for EV separation followed by Western blotting and ELISA for protein analysis, but the low throughput, low efficiency, and high cost of these methods greatly limit their clinical application^164^. Specifically, the clinical monitoring EVs carrying specific ICPs requires more high-purity, low-cost EV separation methods. Current mainstream EV separation techniques include size-based separation, ultracentrifugation, immunoaffinity capture, precipitation methods, microfluidics-based isolation techniques, filtration, or their combinations^165^. Among these, immunoaffinity capture is particularly favored for clinical research because of its direct specificity for distinguishing EVs expressing or carrying ICPs. Although antibody-based immunoaffinity capture methods may occupy antigenic sites on EV surfaces, affecting further analysis, and the risk of nonspecific antibody binding cannot be ignored, it remains a viable option for quantifying PD-L1-EVs. For example, anti-PD-L1 antibody-conjugated gold nanorods attached to EVs produce varying scattering intensities in localized surface plasmon resonance (LSPR)-based nanoplasmonic biosensors based on the expression levels of PD-L1 on each EV; this approach enabled not only semiquantification of total PD-L1-EVs in samples but also differentiation of PD-L1 content on individual EV^166^. The active development of amplifiers for LSPR PD-L1-EV signals is also underway^167^.

However, the detection of PD-L1-EVs in this manner provides information about only the PD-L1 levels of EVs from all cellular sources in patients, including a significant proportion of physiologically released PD-L1-EVs; this limitation reduces the accuracy of this assessment. Researchers have designed a process specifically for patients with melanoma that relies on aptamers to recognize tumor cell-derived PD-L1-EVs. Aptamers are short nucleotide ligands that are smaller than conventional antibodies, allowing easier engineering. Additionally, glycosylated PD-L1, which may be less readily identified by antibodies, can still be recognized by aptamers^168^. The designed aptamers bind to PD-L1 on the surface of EVs and the tumor marker EpCAM. Subsequently, aptamers that bind to different proteins on the same membrane are linked, and with the assistance of a proximity ligation assay, tumor cell-derived PD-L1-EVs can be quantified using droplet digital PCR. This process has been shown to provide greater accuracy in patients with melanoma and may have better potential for predicting responses to ICB therapy^169^. To further reduce the sample volume requirement for quantitative detection with aptamers, another group of researchers combined two types of aptamer probes, one that binds to PD-L1 on the surface of EVs and one that binds to EV-derived miR-21 in EV lysis fluid, using gold nanorods. The addition of two fuel strands mediates probe binding and release, allowing a dual probe to repeatedly bind and release fluorescence groups with PD-L1-EVs and miR-21, thus amplifying the signal for biomarker detection^170^. A novel approach involves attaching the PD-L1 aptamer to ternary metal-metalloid palladium-copper-boron alloy microporous nanospheres that can conduct electrical signals, allowing the detection of PD-L1-EV levels based on characteristic electrical signals and further enhancing the sensitivity of diagnosis in patients with non-small cell lung cancer^171^.

The aforementioned immunological affinity capture strategy still relies primarily on ultracentrifugation for the initial EV purification step. Although this method is considered the gold standard for purifying EVs in current clinical practice, its complexity and high cost limit further dissemination^165^. Magnetic beads with immunological affinity represent a potential alternative, allowing EVs to be captured via their direct adhesion to EVs. Researchers have designed TiO_2_ magnetic nanoparticles that bind indiscriminately to the hydrophilic phosphate heads of exosomal phospholipids, followed by the addition of the PD-L1 marker “Au@Ag@MBA”, which is required for surface-enhanced Raman scattering (SERS) immunoassays; this approach allows the precise quantification of PD-L1-EVs from only 4 μl of a plasma sample^172^. A recent study^173^ introduced artificial Hoogsteen hydrogen bonding interactions to form triple-helix molecular probes (THMPs), which produce a fluorescent signal upon binding with PD-L1. However, magnetic bead-based separation strategies also have considerable limitations, such as high costs and poor reproducibility, necessitating further optimization. Interestingly, one study explored the relationship between PD-L1-EVs derived from the gut microbiome and the response of patients with melanoma to ICB therapy, yielding positive results^174^. This finding highlights the diverse sources of EVs that carry PD-L1, which has great potential to meet various clinical needs. Additionally, the levels of circulating PD-L1-EVs in patients with early osteosarcoma were found to be correlated with long-term prognosis, offering a new perspective for monitoring this cancer type, which lacks effective prognostic markers^175^.

The exploration of the potential of other ICPs as biomarkers is currently in a very early stage. However, some studies have indicated that the levels of CTLA-4, LAG-3, and TIM-3 originating from cells in patient tumor tissues are associated with survival times post-ICB treatment^176,177^. Furthermore, high levels of TIM3-EVs and Gal-9-EVs in the plasma of patients with non-small cell lung cancer are clearly correlated with poor prognosis, suggesting a promising start^178^. Notably, high levels of ubiquitin-like with PHD and ring finger domain 1 RNA within the plasma EVs of patients with hepatocellular carcinoma are associated with a low response to anti-PD-1 therapy, as it degrades miR-449c-5p, thereby upregulating TIM-3 expression in NK cells^179^. The observation of high levels of PD-1-EVs in patients who are resistant to ICB therapy underscores the clinical significance of this research area^82^. The level of FGL1, the ligand for LAG-3, in plasma EVs are related to low responsiveness to PD-1 therapy, a phenomenon not observed when total FGL1 levels are measured^126^. This correlation between EV contents and ICP levels further expands the pool of potential biomarkers for predicting ICB therapy response.

### The Role of EVs in Increasing ICB Treatment Efficacy

**Fig. 2:**
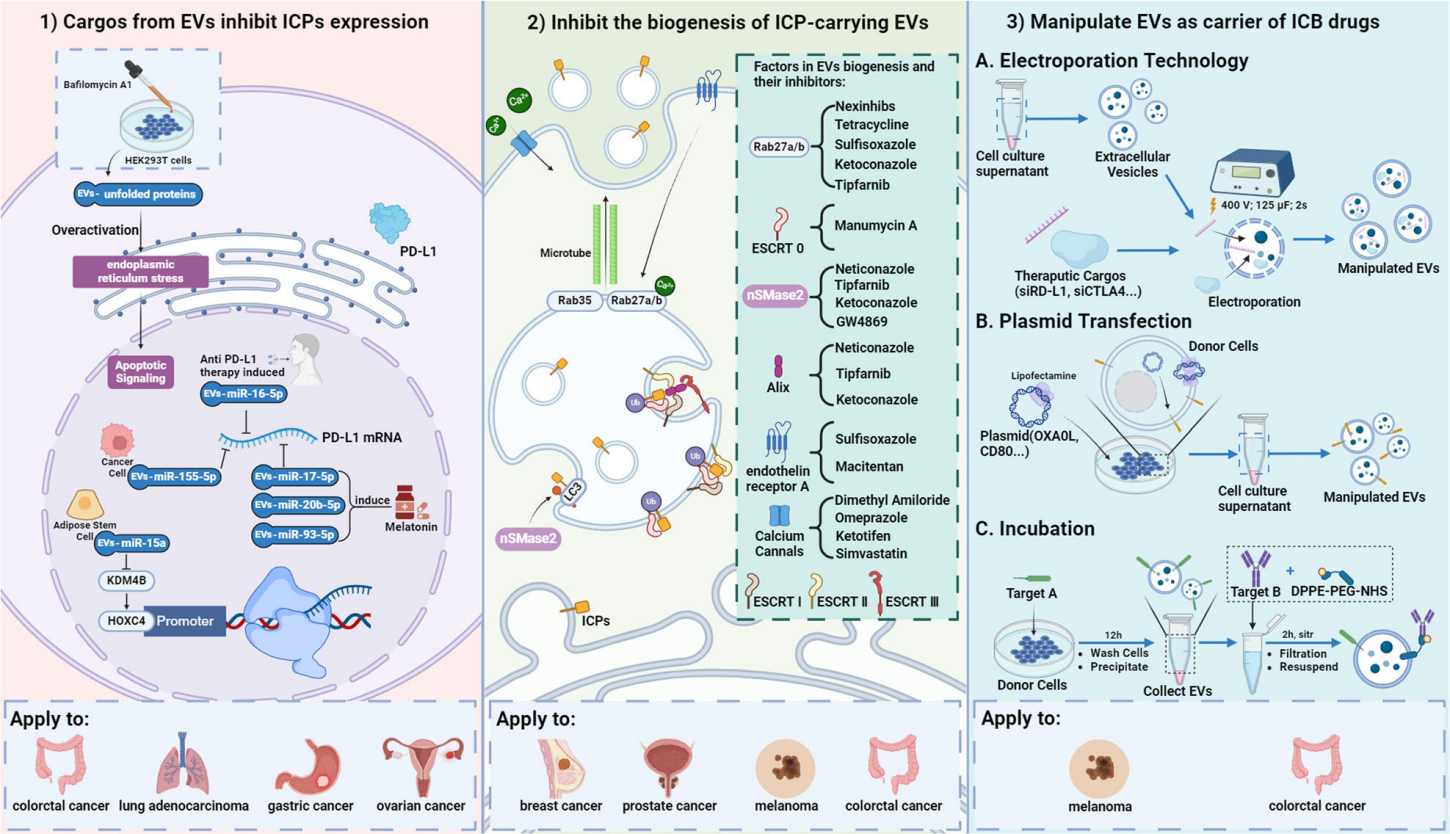
Strategic Interventions in Cancer Therapy Targeting ICP-Related EVs. (1) Tumor microenvironment-derived EVs from various cellular origins can carry cargoes that suppress PD-L1 expression, and these cargoes are packaged into EVs either autonomously or in response to external regulatory influences. (2) The biogenesis of ICP-carrying EVs can be inhibited by targeting Rab27a/b, nSMase2, Alix, endothelin receptor A, and specific inhibitors of calcium channels, affecting their roles in cancer progression and immune evasion. (3) EVs can be manipulated to carry ICP-targeting drugs through electroporation, plasmid transfection, and incubation to deliver these therapeutic agents. These approaches have shown promising results in treating various cancer types, including melanoma and colorectal, breast, and prostate cancers.

Monoclonal anti-ICP antibodies remain the most common clinical treatment to date. However, their instability has prompted researchers to explore other potential ICB alternatives. In particular, given the close relationship between EVs and ICPs, the exploration of ICB alternatives from the perspective of EVs is increasingly gaining attention from researchers (Fig. 2). As we summarized in our previous article^12^, EVs have a bidirectional effect on tumor progression due to the diversity of their cargo, a phenomenon that also occurs in the interaction between EVs and ICPs. In colorectal cancer, EVs derived from adipose mesenchymal stem cells carry miR-15a, which targets lysine demethylase 4B (KDM4B) and inhibits PD-L1 expression by downregulating the binding of homeobox C4 (HOXC4) to the PD-L1 promoter^180^. The expression of miR-16-5p derived from the serum EVs of patients with lung adenocarcinoma who were treated with anti-PD-L1 antibodies increased and could further inhibit PD-L1 expression^181^. The artificial administration of melatonin elevated the levels of several miRNAs in gastric cancer cell-derived EVs that inhibit macrophage PD-L1 expression^22^. Additionally, EV cargo influences the transcription of PD-L1 through the PI3K/Akt and STAT1/3 pathways. Conversely, downregulating these pathways significantly inhibits ICPs on the cell and EV surfaces^29,182^. Designing inhibitors that target miRNAs carried by EVs that regulate PD-L1 has also shown promising results in both cell and animal experiments. Although excessive accumulation of ROS inhibits M2 macrophage uptake of PD-L1-inhibitory EVs, neutralization of ROS with N-acetyl-L-cysteine (NAC) increases the levels of miR-155-5p in tumor-derived EVs that are taken up by macrophages^43^. Interestingly, when cells cannot endure endoplasmic reticulum stress, immunogenic cell death is initiated in tumor cells, and they release tumor antigens and damage-associated molecular pattern (DAMP) to activate surrounding immune cells. When cells were with bafilomycin A1 (bafA1) and EVs carrying misfolded proteins were collected, the induction of endoplasmic reticulum stress in tumor cells after EV uptake caused tumor cell death and induced T-cell activation, significantly enhancing the therapeutic effect when combined with anti-PD-1 treatment^183^.

Direct modulation of the production and release processes of PD-L1-carrying EVs has also shown promise for clinical application (Fig. 2). For example, the use of sulfisoxazole to downregulate Rab27a expression led to a decrease in PD-L1 levels in EVs^182^. GW4869, which blocks EV secretion by inhibiting nSMase synthesis, synergizes with anti-PD-L1 therapy in 4T1 breast cancer-bearing mice^58^. Endothelin receptor A (ETA) is a member of the GPCR family and is extensively involved in the regulation of proteins related to MVB maturation, the fusion of MVBs with the plasma membrane, and autophagic lysosomal degradation^184^. Macitentan, an antimicrobial drug that targets ETA, can also decrease the ability of tumor cells to produce PD-L1-EVs. In addition, it inhibits the capacity of PD-L1 on the surface of EVs to bind with PD-1 on target cells^185^. Additionally, directly disrupting the functionality of the Golgi apparatus in tumor cells has shown to potently suppress the production of PD-L1-EVs^186^. Several pharmacologically potent substances, including tipifarnib, neticonazole, climbazole, ketoconazole, triademenol, manumycin A, and nexinhibs, have shown to inhibit exosome production by reducing the expression of proteins that are essential for ESCRT-dependent exosome formation and the Rab27a transport mechanism^55,88^. Moreover, inhibitors of exosomes that specifically target the ESCRT-independent enzyme N-sphingomyelinase (nSMase), such as GW4869 and spiroepoxide, can block the release of exosomes^187,188^. Despite the current lack of research connecting these drugs with PD-L1-related therapies, this direction has considerable potential.

Owing to the nonimmunogenicity, nontoxicity, and degradation resistance of EVs, they are widely used as drug delivery carriers (Fig. 2). The binding of siRNAs to target genes induces sequence-specific degradation, and the protocol of loading siRNAs into EVs through electroporation and introducing them into organisms has shown high efficiency and safety. Exosomes loaded with PD-L1 siRNA or CTLA-4 siRNA precisely reduce the expression of PD-L1 or CTLA-4 in CRC cells^23^. Loading CD38 siRNA into EVs derived from bone marrow mesenchymal stem cells, which are then taken up by liver cancer cells, downregulates CD38 enzyme activity and adenosine secretion, thereby promoting the differentiation of macrophages toward the M1 phenotype and reducing the release of PD-L1-EVs^189^. Additionally, researchers have extracted EVs carrying large amounts of target proteins from plasmids to assist in ICB therapy. For example, EVs from epithelial cells carry a large amount of OX40L, which, by activating the function of CD4^+^ T cells, further enhances the therapeutic effect against CTLA-4^24^.

Recent research has revealed that increasing the activity of tumor-specific immune cells within the human body can significantly increase the efficacy of ICB therapy^190^. The interaction between these cells and EVs has been found to further amplify this effect. Dendritic cells (DCs) that are educated by tumor-derived EVs promote the proliferation and function of tumor-specific T cells. Moreover, these cells can release EVs carrying tumor-specific antigens, which, when ingested by tumor cells, increase the immunogenicity of tumor cells. When these EVs bind to T cells, they can promote T-cell activation and function. Although the function of T cells is eventually exhausted by PD-L1 in the tumor microenvironment, the significantly increased baseline number of T cells results in a significant increase in the number of T cells that regain activity after anti-PD-L1 treatment^191,192^. When reinfused into mice, tumor-specific neoantigen peptides that are carried by serum EVs from mice are efficiently taken upby dendritic cells, in turn inducing the production of more specific T cells and further facilitating anti-PD-1 therapy^193^. Additionally, because tumor-activated dendritic cells migrate to lymph nodes and dendritic cell-derived EVs are able to home to their parent cells, subcutaneous injections of dendritic cell-derived EVs carrying tumor antigens rapidly accumulate in tumor-draining lymph nodes^194^. By using lipid anchoring technology to modify anti-CTLA4 antibodies on the surface of DC-derived EVs, these EVs can efficiently bind to receptors on the surface of CD4^+^ T cells in lymph nodes. These EVs rely on antigens, MHC molecules, and CD80 on their surface to activate T cells while also blocking the transmission of T-cell inhibitory signals^195^.

## Limitations in ICP-Related EV Research

Despite substantial advances in the research of EVs and ICPs over the past few decades, studies that consider them as functionally interacting factors remain insufficient. The complex network of EV‒ICP interactions remains a field with many unanswered questions, especially for newly discovered ICPs such as CTLA4, TIM3, LAG3, and ICOS. Although the roles of ICPs in intercellular regulatory processes have been partially elucidated, their presence in EVs has not received the same level of attention as that of PD-L1. Given the diversity of EV sources and their unique mechanisms of action, it is plausible that ICPs associated with EVs may perform distinct and unexpected functions. Further investigations into these various ICPs in EVs are warranted and may significantly expand the horizons of tumor immunotherapy.

With advances in EV isolation and identification technologies, the theoretical feasibility of using ICPs as biomarkers for cancer diagnosis and prognosis is increasing. The diversity and programmability of EV cargo also endow them with potential as mediators for ICB therapy. However, these diagnostic and monitoring methods lack long-term observational results, and their efficacy in practical situations remains uncertain. Additionally, most researches on the reprogramming of EVs for tumor treatment are limited to cellular or animal models; studies on the use of this therapy within the complex human tumor microenvironment seem premature. Another technical bottleneck of such engineered EVs is the lack of methods to efficiently and consistently obtain EVs that meet the requirements of clinical practice. Nevertheless, researchers have never ceased their efforts to translate EVs into clinical applications. For example, a recent study discussed the trade-offs involved in developing therapeutic methods using mesenchymal stem cell-derived EVs^196^.

## Conclusion

In this review, we explore the complex interactions between EVs and key ICPs, including PD-L1, CTLA4, TIM3, LAG3, and ICOS. EVs play a crucial role in the lifecycle of these ICPs, influencing their production, transport, and functionality. EVs not only carry cargo that can modulate ICP expression but also facilitate the transfer of ICP ligands and receptors between cells, inducing complex regulatory mechanisms and altering the biological properties of EVs to further tumor progression. This interplay presents a sophisticated functional landscape of ICPs and opens new avenues for their clinical application.

## Data Availability

Data sharing is not applicable to this article, as no datasets were generated or analyzed during the current study.

## References

[1] Linsley, P. S. et al. CTLA-4 is a second receptor for the B cell activation antigen B7. *J. Exp. Med.* **174**, 561–569 (1991). 1714933 10.1084/jem.174.3.561 PMC2118936

[2] Xing, C. et al. The roles of exosomal immune checkpoint proteins in tumors. *Mil. Med. Res.* **8**, 56 (2021). 34743730 10.1186/s40779-021-00350-3 PMC8573946

[3] Gaikwad, S., Agrawal, M. Y., Kaushik, I., Ramachandran, S. & Srivastava, S. K. Immune checkpoint proteins: Signaling mechanisms and molecular interactions in cancer immunotherapy. *Semin. Cancer Biol.* **86**, 137–150 (2022). 35341913 10.1016/j.semcancer.2022.03.014

[4] Weber, J. Immune checkpoint proteins: a new therapeutic paradigm for cancer-preclinical background: CTLA-4 and PD-1 blockade. *Semin. Oncol.* **37**, 430–439 (2010). 21074057 10.1053/j.seminoncol.2010.09.005

[5] He, X. & Xu, C. Immune checkpoint signaling and cancer immunotherapy. *Cell Res.* **30**, 660–669 (2020). 32467592 10.1038/s41422-020-0343-4 PMC7395714

[6] Ribas, A. & Wolchok, J. D. Cancer immunotherapy using checkpoint blockade. *Sci. (N. Y., NY)* **359**, 1350–1355 (2018). 10.1126/science.aar4060 PMC7391259 29567705

[7] Hodi, F. S. et al. Improved survival with ipilimumab in patients with metastatic melanoma. *N. Engl. J. Med.* **363**, 711–723 (2010). 20525992 10.1056/NEJMoa1003466 PMC3549297

[8] Beaver, J. A. et al. Patients with melanoma treated with an anti-PD-1 antibody beyond RECIST progression: a US Food and Drug Administration pooled analysis. *Lancet Oncol.* **19**, 229–239 (2018). 29361469 10.1016/S1470-2045(17)30846-X PMC5806609

[9] Heidegger, S. et al. Targeting nucleic acid sensors in tumor cells to reprogram biogenesis and RNA cargo of extracellular vesicles for T cell-mediated cancer immunotherapy. *Cell Rep. Med.* **4**, 101171 (2023). 37657445 10.1016/j.xcrm.2023.101171 PMC10518594

[10] Wang, A. et al. Pyroptosis and the tumor immune microenvironment: A new battlefield in ovarian cancer treatment. Biochimica et biophysica acta Reviews on cancer. 2023:189058. 10.1016/j.bbcan.2023.189058 38113952

[11] Fang, J. et al. Exploring the crosstalk between endothelial cells, immune cells, and immune checkpoints in the tumor microenvironment: new insights and therapeutic implications. *Cell Death Dis.* **14**, 586 (2023). 37666809 10.1038/s41419-023-06119-x PMC10477350

[12] Ye, Z., Chen, W., Li, G., Huang, J., Lei, J. Tissue-derived extracellular vesicles in cancer progression: mechanisms, roles, and potential applications. *Cancer Metastasis Rev.* (2023). 10.1007/s10555-023-10147-6 37851319

[13] Théry, C. et al. Minimal information for studies of extracellular vesicles 2018 (MISEV2018): a position statement of the International Society for Extracellular Vesicles and update of the MISEV2014 guidelines. *J. Extracell. vesicles* **7**, 1535750 (2018). 30637094 10.1080/20013078.2018.1535750 PMC6322352

[14] Guo, S. et al. The role of extracellular vesicles in circulating tumor cell-mediated distant metastasis. *Mol. Cancer* **22**, 193 (2023). 38037077 10.1186/s12943-023-01909-5 PMC10688140

[15] Yue, M. et al. Extracellular vesicles remodel tumor environment for cancer immunotherapy. *Mol. Cancer* **22**, 203 (2023). 38087360 10.1186/s12943-023-01898-5 PMC10717809

[16] Alia Moosavian, S., Hashemi, M., Etemad, L., Daneshmand, S. & Salmasi, Z. Melanoma-derived exosomes: Versatile extracellular vesicles for diagnosis, metastasis, immune modulation, and treatment of melanoma. *Int. Immunopharmacol.* **113**, 109320 (2022). 36274482 10.1016/j.intimp.2022.109320

[17] Fyfe, J., Dye, D., Razak, N. B. A., Metharom, P. & Falasca, M. Immune evasion on the nanoscale: Small extracellular vesicles in pancreatic ductal adenocarcinoma immunity. *Semin. Cancer Biol.* **96**, 36–47 (2023). 37748738 10.1016/j.semcancer.2023.09.004

[18] Li, L. et al. Exosomes as a modulator of immune resistance in human cancers. *Cytokine Growth Factor Rev.* **73**, 135–149 (2023). 37543438 10.1016/j.cytogfr.2023.07.007

[19] Wang, H. et al. High throughput and noninvasive exosomal PD-L1 detection for accurate immunotherapy response prediction via Tim4-functionalized magnetic core-shell metal-organic frameworks. *Anal. Chem.* **95**, 18268–18277 (2023). 38011622 10.1021/acs.analchem.3c04117

[20] Park, S. J. et al. Clinical significance of serum-derived exosomal PD-L1 expression in patients with advanced pancreatic cancer. *BMC cancer* **23**, 389 (2023). 37127565 10.1186/s12885-023-10811-8 PMC10150468

[21] Xu, P. et al. Clinical significance of plasma PD-L1(+) exosomes in the management of diffuse large B cell lymphoma. *Ann. Hematol.* **102**, 2435–2444 (2023). 37162517 10.1007/s00277-023-05259-6

[22] Wang, K. et al. Melatonin enhances anti-tumor immunity by targeting macrophages PD-L1 via exosomes derived from gastric cancer cells. *Mol. Cell. Endocrinol.* **568-569**, 111917 (2023). 37028587 10.1016/j.mce.2023.111917

[23] Li, J. et al. Exosomes-delivered PD-L1 siRNA and CTLA-4 siRNA protect against growth and tumor immune escape in colorectal cancer. *Genomics* **115**, 110646 (2023). 37217085 10.1016/j.ygeno.2023.110646

[24] Kugeratski, F. G. et al. Engineered immunomodulatory extracellular vesicles derived from epithelial cells acquire capacity for positive and negative T cell co-stimulation in cancer and autoimmunity. bioRxiv : the preprint server for biology. (2023).

[25] Akhtar, M., Rashid, S. & Al-Bozom, I. A. PD-L1 immunostaining: what pathologists need to know. *Diagnostic Pathol.* **16**, 94 (2021). 10.1186/s13000-021-01151-x PMC8543866 34689789

[26] Akinleye, A. & Rasool, Z. Immune checkpoint inhibitors of PD-L1 as cancer therapeutics. *J. Hematol. Oncol.* **12**, 92 (2019). 31488176 10.1186/s13045-019-0779-5 PMC6729004

[27] Chamoto, K., Yaguchi, T., Tajima, M. & Honjo, T. Insights from a 30-year journey: function, regulation and therapeutic modulation of PD1. *Nat. Rev. Immunol.* **23**, 682–695 (2023). 37185300 10.1038/s41577-023-00867-9

[28] Glaviano, A. et al. PI3K/AKT/mTOR signaling transduction pathway and targeted therapies in cancer. *Mol. Cancer* **22**, 138 (2023). 37596643 10.1186/s12943-023-01827-6 PMC10436543

[29] Xu, W., Chen, Y., Zhang, Z., Jiang, Y. & Wang, Z. Exosomal PIK3CB promotes PD-L1 expression and malignant transformation in esophageal squamous cell carcinoma. *Med. Oncol. (Northwood, Lond., Engl.)* **40**, 221 (2023). 10.1007/s12032-023-02093-8 37402056

[30] Xu, D. et al. Tumor-derived small extracellular vesicles promote breast cancer progression by upregulating PD-L1 expression in macrophages. *Cancer cell Int.* **23**, 137 (2023). 37452413 10.1186/s12935-023-02980-0 PMC10347751

[31] Zhao, L. et al. LOXL4 shuttled by tumor cells-derived extracellular vesicles promotes immune escape in hepatocellular carcinoma by activating the STAT1/PD-L1 Axis. *J. Immunother. (Hagerstown, Md : 1997)* **47**, 64–76 (2024). 10.1097/CJI.0000000000000496 38047403

[32] Han, N., Zhou, D., Ruan, M., Yan, M. & Zhang, C. Cancer cell-derived extracellular vesicles drive pre-metastatic niche formation of lymph node via IFNGR1/JAK1/STAT1-activated-PD-L1 expression on FRCs in head and neck cancer. *Oral. Oncol.* **145**, 106524 (2023). 37482043 10.1016/j.oraloncology.2023.106524

[33] Lütge, M., Pikor, N. B. & Ludewig, B. Differentiation and activation of fibroblastic reticular cells. *Immunological Rev.* **302**, 32–46 (2021). 10.1111/imr.12981 PMC8361914 34046914

[34] Yin, L. & Wang, Y. Extracellular vesicles derived from M2-polarized tumor-associated macrophages promote immune escape in ovarian cancer through NEAT1/miR-101-3p/ZEB1/PD-L1 axis. *Cancer Immunol., Immunother. : CII* **72**, 743–758 (2023). 36319716 10.1007/s00262-022-03305-2 PMC10992138

[35] Wang, Q. M. et al. Exosomal lncRNA NEAT1 inhibits NK cell activity to promote multiple myeloma cell immune escape via an EZH2/PBX1 axis. *Mol. Cancer Res*. (2023). 10.1158/1541-7786.MCR-23-0282 37889101

[36] Yang, K., Zhang, J. & Bao, C. Exosomal circEIF3K from cancer-associated fibroblast promotes colorectal cancer (CRC) progression via miR-214/PD-L1 axis. *BMC Cancer* **21**, 933 (2021). 34412616 10.1186/s12885-021-08669-9 PMC8375187

[37] Zhao, B. et al. Lnc-CCNH-8 promotes immune escape by up-regulating PD-L1 in hepatocellular carcinoma. *Mol. Ther. Nucleic acids* **35**, 102125 (2024). 38356866 10.1016/j.omtn.2024.102125 PMC10865404

[38] Cubillos-Ruiz, J. R., Bettigole, S. E. & Glimcher, L. H. Tumorigenic and immunosuppressive effects of endoplasmic reticulum stress in cancer. *Cell* **168**, 692–706 (2017). 28187289 10.1016/j.cell.2016.12.004 PMC5333759

[39] Fu, X. et al. Endoplasmic reticulum stress, cell death and tumor: association between endoplasmic reticulum stress and the apoptosis pathway in tumors (Review). *Oncol. Rep.* **45**, 801–808 (2021). 33469681 10.3892/or.2021.7933 PMC7859917

[40] Yuan, Y. et al. Endoplasmic reticulum stress promotes the release of exosomal PD-L1 from head and neck cancer cells and facilitates M2 macrophage polarization. *Cell Commun. Signal.* **20**, 12 (2022). 35090495 10.1186/s12964-021-00810-2 PMC8796490

[41] Yan, D., Wang, H. W., Bowman, R. L. & Joyce, J. A. STAT3 and STAT6 signaling pathways synergize to promote cathepsin secretion from macrophages via IRE1α activation. *Cell Rep.* **16**, 2914–2927 (2016). 27626662 10.1016/j.celrep.2016.08.035 PMC5559199

[42] Liu, J. et al. Endoplasmic reticulum stress causes liver cancer cells to release exosomal miR-23a-3p and up-regulate programmed death ligand 1 expression in macrophages. *Hepatol. (Baltim., Md)* **70**, 241–258 (2019). 10.1002/hep.30607 PMC6597282 30854665

[43] Li, X. et al. Reactive oxygen species reprogram macrophages to suppress antitumor immune response through the exosomal miR-155-5p/PD-L1 pathway. *J. Exp. Clin. cancer Res.* **41**, 41 (2022). 35086548 10.1186/s13046-022-02244-1 PMC8793215

[44] Yuan, Y. et al. Exosomal O-GlcNAc transferase from esophageal carcinoma stem cell promotes cancer immunosuppression through up-regulation of PD-1 in CD8(+) T cells. *Cancer Lett.* **500**, 98–106 (2021). 33307156 10.1016/j.canlet.2020.12.012

[45] Liu, J. et al. Extracellular vesicle PD-L1 in reshaping tumor immune microenvironment: biological function and potential therapy strategies. *Cell Commun. Signal.* **20**, 14 (2022). 35090497 10.1186/s12964-021-00816-w PMC8796536

[46] Hsu, J. M. et al. STT3-dependent PD-L1 accumulation on cancer stem cells promotes immune evasion. *Nat. Commun.* **9**, 1908 (2018). 29765039 10.1038/s41467-018-04313-6 PMC5954021

[47] Okada, M. et al. Blockage of core fucosylation reduces cell-surface expression of PD-1 and promotes anti-tumor immune responses of T cells. *Cell Rep.* **20**, 1017–1028 (2017). 28768188 10.1016/j.celrep.2017.07.027

[48] Li, C. W. et al. Glycosylation and stabilization of programmed death ligand-1 suppresses T-cell activity. *Nat. Commun.* **7**, 12632 (2016). 27572267 10.1038/ncomms12632 PMC5013604

[49] Zhu, L. et al. Quantification-promoted discovery of glycosylated exosomal PD-L1 as a potential tumor biomarker. *Small methods* **6**, e2200549 (2022). 35810463 10.1002/smtd.202200549

[50] Zhu, L. et al. Coupling aptamer-based protein tagging with metabolic glycan labeling for in situ visualization and biological function study of exosomal protein-specific glycosylation. *Angew. Chem. (Int. ed. Engl.)* **60**, 18111–18115 (2021). 34043264 10.1002/anie.202103696

[51] Sun, W. et al. Tumor stem cell-derived exosomal microRNA-17-5p inhibits anti-tumor immunity in colorectal cancer via targeting SPOP and overexpressing PD-L1. *Cell death Discov.* **8**, 223 (2022). 35461336 10.1038/s41420-022-00919-4 PMC9035163

[52] Gou, Q. et al. PD-L1 degradation pathway and immunotherapy for cancer. *Cell death Dis.* **11**, 955 (2020). 33159034 10.1038/s41419-020-03140-2 PMC7648632

[53] Xian, D., Niu, L., Zeng, J. & Wang, L. LncRNA KCNQ1OT1 secreted by tumor cell-derived exosomes mediates immune escape in colorectal cancer by regulating PD-L1 Ubiquitination via MiR-30a-5p/USP22. *Front. cell developmental Biol.* **9**, 653808 (2021). 10.3389/fcell.2021.653808 PMC8326752 34350172

[54] Jing, H. et al. Integrin α2 promotes immune escape in non-small-cell lung cancer by enhancing PD-L1 expression in exosomes to inhibit CD8 + T-cell activity. *J. investigative Med. : Off. Publ. Am. Federation Clin. Res.* **72**, 57–66 (2024). 10.1177/10815589231207801 37804164

[55] Alam, M. R., Rahman, M. M. & Li, Z. The link between intracellular calcium signaling and exosomal PD-L1 in cancer progression and immunotherapy. *Genes Dis.* **11**, 321–334 (2024). 37588227 10.1016/j.gendis.2023.01.026 PMC10425812

[56] Wei, F. et al. Exosomal PD-L1 derived from head and neck squamous cell carcinoma promotes immune evasion by activating the positive feedback loop of activated regulatory T cell-M2 macrophage. *Oral. Oncol.* **145**, 106532 (2023). 37499326 10.1016/j.oraloncology.2023.106532

[57] Tang, Y. et al. The biogenesis, biology, and clinical significance of exosomal PD-L1 in cancer. *Front. Immunol.* **11**, 604 (2020). 32322256 10.3389/fimmu.2020.00604 PMC7158891

[58] Yang, Y. et al. Exosomal PD-L1 harbors active defense function to suppress T cell killing of breast cancer cells and promote tumor growth. *Cell Res.* **28**, 862–864 (2018). 29959401 10.1038/s41422-018-0060-4 PMC6082826

[59] Xu, R. et al. Extracellular vesicles in cancer - implications for future improvements in cancer care. *Nat. Rev. Clin. Oncol.* **15**, 617–638 (2018). 29795272 10.1038/s41571-018-0036-9

[60] Chen, J. et al. GOLM1 exacerbates CD8(+) T cell suppression in hepatocellular carcinoma by promoting exosomal PD-L1 transport into tumor-associated macrophages. *Signal Transduct. Target. Ther.* **6**, 397 (2021). 34795203 10.1038/s41392-021-00784-0 PMC8602261

[61] Ye, Z. et al. Manipulation of PD-L1 endosomal trafficking promotes anticancer immunity. *Adv. Sci. (Weinh., Baden.-Wurtt., Ger.)* **10**, e2206411 (2023). 10.1002/advs.202206411 PMC9951344 36567273

[62] Shen, D. D. et al. LSD1 deletion decreases exosomal PD-L1 and restores T-cell response in gastric cancer. *Mol. cancer* **21**, 75 (2022). 35296335 10.1186/s12943-022-01557-1 PMC8925194

[63] Gu, H. et al. Sorting protein VPS33B regulates exosomal autocrine signaling to mediate hematopoiesis and leukemogenesis. *J. Clin. Investig.* **126**, 4537–4553 (2016). 27797340 10.1172/JCI87105 PMC5127665

[64] Villarroya-Beltri, C. et al. ISGylation controls exosome secretion by promoting lysosomal degradation of MVB proteins. *Nat. Commun.* **7**, 13588 (2016). 27882925 10.1038/ncomms13588 PMC5123068

[65] Kanemoto, S. et al. Multivesicular body formation enhancement and exosome release during endoplasmic reticulum stress. *Biochemical biophysical Res. Commun.* **480**, 166–172 (2016). 10.1016/j.bbrc.2016.10.019 27725157

[66] Gurung, S., Perocheau, D., Touramanidou, L. & Baruteau, J. The exosome journey: from biogenesis to uptake and intracellular signalling. *Cell Commun. Signal. : CCS* **19**, 47 (2021). 33892745 10.1186/s12964-021-00730-1 PMC8063428

[67] Liu, D. A. et al. A phosphoinositide switch mediates exocyst recruitment to multivesicular endosomes for exosome secretion. *Nat. Commun.* **14**, 6883 (2023). 37898620 10.1038/s41467-023-42661-0 PMC10613218

[68] Xiang, J. et al. Exo70 Promotes the invasion of pancreatic cancer cells via the regulation of exosomes. *Cancers* **16** (2024). 10.3390/cancers16020336 PMC10813805 38254825

[69] Izquierdo, E. et al. Extracellular vesicles and PD-L1 suppress macrophages, inducing therapy resistance in TP53-deficient B-cell malignancies. *Blood* **139**, 3617–3629 (2022). 35344582 10.1182/blood.2021014007

[70] Yu, X., Harris, S. L. & Levine, A. J. The regulation of exosome secretion: a novel function of the p53 protein. *Cancer Res.* **66**, 4795–4801 (2006). 16651434 10.1158/0008-5472.CAN-05-4579

[71] Wang, J. et al. Helicobacter pylori CagA promotes immune evasion of gastric cancer by upregulating PD-L1 level in exosomes. *iScience* **26**, 108414 (2023). 38047083 10.1016/j.isci.2023.108414 PMC10692710

[72] Qiu, Y. et al. Activated T cell-derived exosomal PD-1 attenuates PD-L1-induced immune dysfunction in triple-negative breast cancer. *Oncogene* **40**, 4992–5001 (2021). 34172932 10.1038/s41388-021-01896-1 PMC8342306

[73] Xu, R. et al. Aptamer-Assisted Traceless Isolation of PD-L1-Positive Small Extracellular Vesicles for Dissecting Their Subpopulation Signature and Function. *Anal. Chem.* **95**, 1016–1026 (2023). 36534080 10.1021/acs.analchem.2c03725

[74] Wang, J. et al. Exosomal PD-L1 and N-cadherin predict pulmonary metastasis progression for osteosarcoma patients. *J. nanobiotechnology* **18**, 151 (2020). 33092576 10.1186/s12951-020-00710-6 PMC7579953

[75] Lin, W. et al. Extracellular vesicle-cell adhesion molecules in tumours: biofunctions and clinical applications. *Cell Commun. Signal. : CCS* **21**, 246 (2023). 37735659 10.1186/s12964-023-01236-8 PMC10512615

[76] Zhang, W. et al. ICAM-1-mediated adhesion is a prerequisite for exosome-induced T cell suppression. *Developmental cell* **57**, 329–43.e7 (2022). 35085484 10.1016/j.devcel.2022.01.002 PMC8881799

[77] Wang, R., Yang, Y., Huang, J. & Yao, Y. The detection of exosomal PD-L1 in peripheral blood. *Methods Mol. Biol. (Clifton, NJ)* **2695**, 195–212 (2023). 10.1007/978-1-0716-3346-5_13 37450120

[78] Feng, R. et al. Cancer-associated fibroblast-derived extracellular vesicles mediate immune escape of bladder cancer via PD-L1/PD-1 expression. *Endocr. Metab. immune Disord. drug targets* **23**, 1410–1420 (2023). 36852791 10.2174/1871530323666230228124125

[79] Yu, Z. L., Liu, J. Y. & Chen, G. Small extracellular vesicle PD-L1 in cancer: the knowns and unknowns. *NPJ Precis. Oncol.* **6**, 42 (2022). 35729210 10.1038/s41698-022-00287-3 PMC9213536

[80] Ko, H. H. et al. Metastasis and immunosuppression promoted by mtDNA and PD-L1 in extracellular vesicles are reversed by WGP β-glucan in oral squamous cell carcinoma. *Cancer Sci.* **114**, 3857–3872 (2023). 37525561 10.1111/cas.15919 PMC10551585

[81] Ou, Q., Dou, X., Tang, J., Wu, P. & Pan, D. Small extracellular vesicles derived from PD-L1-modified mesenchymal stem cell promote Tregs differentiation and prolong allograft survival. *Cell tissue Res.* **389**, 465–481 (2022). 35688948 10.1007/s00441-022-03650-9

[82] Wang, B. et al. Mutual regulation of PD-L1 immunosuppression between tumor-associated macrophages and tumor cells: a critical role for exosomes. *Cell Commun. Signal.* **22**, 21 (2024). 38195554 10.1186/s12964-024-01473-5 PMC10775441

[83] Schroeder, J. C. et al. Circulating exosomes inhibit B cell proliferation and activity. *Cancers* **12** (2020). 10.3390/cancers12082110 PMC7464446 32751214

[84] Chen, Y. et al. Jianpi Yangzheng Xiaozheng decoction alleviates gastric cancer progression via suppressing exosomal PD-L1. *Front. Pharmacol.* **14**, 1159829 (2023). 37601051 10.3389/fphar.2023.1159829 PMC10434994

[85] Poggio, M. et al. Suppression of Exosomal PD-L1 Induces Systemic Anti-tumor Immunity and Memory. *Cell* **177**, 414–27.e13 (2019). 30951669 10.1016/j.cell.2019.02.016 PMC6499401

[86] Li, D. et al. Prostate cancer cells synergistically defend against CD8(+) T cells by secreting exosomal PD-L1. *Cancer Med.* **12**, 16405–16415 (2023). 37501397 10.1002/cam4.6275 PMC10469662

[87] Ma, J., Cen, Q., Wang, Q., Liu, L. & Zhou, J. Exosomes released from PD-L1(+) tumor associated macrophages promote peritoneal metastasis of epithelial ovarian cancer by up-regulating T cell lipid metabolism. *Biochem. biophysics Rep.* **36**, 101542 (2023). 10.1016/j.bbrep.2023.101542 PMC10563010 37822876

[88] Yin, Z. et al. Mechanisms underlying low-clinical responses to PD-1/PD-L1 blocking antibodies in immunotherapy of cancer: a key role of exosomal PD-L1. Journal for immunotherapy of cancer. 2021;9. 10.1136/jitc-2020-001698 PMC7818841 33472857

[89] Chen, G. et al. Exosomal PD-L1 contributes to immunosuppression and is associated with anti-PD-1 response. *Nature* **560**, 382–386 (2018). 30089911 10.1038/s41586-018-0392-8 PMC6095740

[90] Theodoraki, M. N., Yerneni, S. S., Hoffmann, T. K., Gooding, W. E. & Whiteside, T. L. Clinical Significance of PD-L1(+) Exosomes in Plasma of Head and Neck Cancer Patients. *Clin. Cancer Res. : Off. J. Am. Assoc. Cancer Res.* **24**, 896–905 (2018). 10.1158/1078-0432.CCR-17-2664 PMC6126905 29233903

[91] Chen, J. et al. Tumor extracellular vesicles mediate anti-PD-L1 therapy resistance by decoying anti-PD-L1. *Cell. Mol. Immunol.* **19**, 1290–1301 (2022). 36220994 10.1038/s41423-022-00926-6 PMC9622748

[92] Gong, B. et al. Secreted PD-L1 variants mediate resistance to PD-L1 blockade therapy in non-small cell lung cancer. *J. Exp. Med.* **216**, 982–1000 (2019). 30872362 10.1084/jem.20180870 PMC6446862

[93] Serratì, S. et al. Circulating extracellular vesicles expressing PD1 and PD-L1 predict response and mediate resistance to checkpoint inhibitors immunotherapy in metastatic melanoma. *Mol. cancer* **21**, 20 (2022). 35042524 10.1186/s12943-021-01490-9 PMC8764806

[94] Affolter A, et al. Modulation of PD‑L1 expression by standard therapy in head and neck cancer cell lines and exosomes. International journal of oncology. 2023;63. 10.3892/ijo.2023.5550 PMC10552694 37503786

[95] Zheng, Y. et al. Glioblastoma stem cell (GSC)-derived PD-L1-containing exosomes activates AMPK/ULK1 pathway mediated autophagy to increase temozolomide-resistance in glioblastoma. *Cell Biosci.* **11**, 63 (2021). 33789726 10.1186/s13578-021-00575-8 PMC8011168

[96] Rudd, C. E., Taylor, A. & Schneider, H. CD28 and CTLA-4 coreceptor expression and signal transduction. *Immunological Rev.* **229**, 12–26 (2009). 10.1111/j.1600-065X.2009.00770.x PMC4186963 19426212

[97] Koni, M. et al. Circulating extracellular vesicles derived from tumor endothelial cells hijack the local and systemic anti-tumor immune response: Role of mTOR/G-CSF pathway. *Pharmacol. Res.* **195**, 106871 (2023). 37506784 10.1016/j.phrs.2023.106871

[98] Weng, H. P. et al. Canine diffuse large b-cell lymphoma downregulates the activity of CD8 + T-cells through tumor-derived extracellular vesicles. *Cancer cell Int.* **23**, 252 (2023). 37884996 10.1186/s12935-023-03104-4 PMC10601183

[99] Ukrainskaya, V. M. et al. CAR-tropic extracellular vesicles carry tumor-associated antigens and modulate CAR T cell functionality. *Sci. Rep.* **13**, 463 (2023). 36627334 10.1038/s41598-023-27604-5 PMC9832131

[100] Walker, L. S. K. The link between circulating follicular helper T cells and autoimmunity. *Nat. Rev. Immunol.* **22**, 567–575 (2022). 35277664 10.1038/s41577-022-00693-5 PMC8915145

[101] Li, Z. et al. Esophageal cancer cell-derived small extracellular vesicles decrease circulating Tfh/Tfr via sEV-PDL1 to promote immunosuppression. *Cancer Immunol., Immunother. : CII* **72**, 4249–4259 (2023). 37943341 10.1007/s00262-023-03561-w PMC10992026

[102] Oyewole-Said, D. et al. Beyond T-Cells: Functional Characterization of CTLA-4 Expression in Immune and Non-Immune Cell Types. *Front. Immunol.* **11**, 608024 (2020). 33384695 10.3389/fimmu.2020.608024 PMC7770141

[103] Liu, L. et al. Hepatic stellate cell exosome-derived circWDR25 promotes the progression of hepatocellular carcinoma via the miRNA-4474-3P-ALOX-15 and EMT axes. *Biosci. trends* **16**, 267–281 (2022). 35934785 10.5582/bst.2022.01281

[104] Mondal, S. K., Haas, D., Han, J. & Whiteside, T. L. Small EV in plasma of triple negative breast cancer patients induce intrinsic apoptosis in activated T cells. *Commun. Biol.* **6**, 815 (2023). 37542121 10.1038/s42003-023-05169-3 PMC10403597

[105] Benecke, L. et al. Exosomes: Small EVs with Large Immunomodulatory Effect in Glioblastoma. International journal of molecular sciences. 2021;22. 10.3390/ijms22073600 PMC8036988 33808435

[106] Wang, Y. et al. Exosome CTLA-4 Regulates PTEN/CD44 Signal Pathway in Spleen Deficiency Internal Environment to Promote Invasion and Metastasis of Hepatocellular Carcinoma. *Front. Pharmacol.* **12**, 757194 (2021). 34744733 10.3389/fphar.2021.757194 PMC8564353

[107] Wolf, Y., Anderson, A. C. & Kuchroo, V. K. TIM3 comes of age as an inhibitory receptor. *Nat. Rev. Immunol.* **20**, 173–185 (2020). 31676858 10.1038/s41577-019-0224-6 PMC7327798

[108] Cai, L., Li, Y., Tan, J., Xu, L. & Li, Y. Targeting LAG-3, TIM-3, and TIGIT for cancer immunotherapy. *J. Hematol. Oncol.* **16**, 101 (2023). 37670328 10.1186/s13045-023-01499-1 PMC10478462

[109] Chiba, S. et al. Tumor-infiltrating DCs suppress nucleic acid-mediated innate immune responses through interactions between the receptor TIM-3 and the alarmin HMGB1. *Nat. Immunol.* **13**, 832–842 (2012). 22842346 10.1038/ni.2376 PMC3622453

[110] Ndhlovu, L. C. et al. Tim-3 marks human natural killer cell maturation and suppresses cell-mediated cytotoxicity. *Blood* **119**, 3734–3743 (2012). 22383801 10.1182/blood-2011-11-392951 PMC3335380

[111] Clayton, K. L. et al. T cell Ig and mucin domain-containing protein 3 is recruited to the immune synapse, disrupts stable synapse formation, and associates with receptor phosphatases. *J. Immunol. (Baltim., Md : 1950)* **192**, 782–791 (2014). 10.4049/jimmunol.1302663 PMC4214929 24337741

[112] Lefebvre, A. et al. Extracellular vesicles derived from nasopharyngeal carcinoma induce the emergence of mature regulatory dendritic cells using a galectin-9 dependent mechanism. *J. Extracell. vesicles* **12**, e12390 (2023). 38117000 10.1002/jev2.12390 PMC10731827

[113] de Mingo Pulido, Á. et al. The inhibitory receptor TIM-3 limits activation of the cGAS-STING pathway in intra-tumoral dendritic cells by suppressing extracellular DNA uptake. *Immunity* **54**, 1154–67.e7 (2021). 33979578 10.1016/j.immuni.2021.04.019 PMC8192496

[114] Zhang, C. X. et al. Galectin-9 promotes a suppressive microenvironment in human cancer by enhancing STING degradation. *Oncogenesis* **9**, 65 (2020). 32632113 10.1038/s41389-020-00248-0 PMC7338349

[115] Wu, J. et al. Exosomes in malignant pleural effusion from lung cancer patients impaired the cytotoxicity of double-negative T cells. *Transl. Oncol.* **27**, 101564 (2023). 36252282 10.1016/j.tranon.2022.101564 PMC9579705

[116] Javadi, J. et al. Diagnostic and prognostic utility of the extracellular vesicles subpopulations present in pleural effusion. *Biomolecules*. **11** (2021). 10.3390/biom11111606 PMC8615485 34827604

[117] Li, X., Liu, Y., Yang, L., Jiang, Y. & Qian, Q. TIM-3 shuttled by MV3 cells-secreted exosomes inhibits CD4(+) T cell immune function and induces macrophage M2 polarization to promote the growth and metastasis of melanoma cells. *Transl. Oncol.* **18**, 101334 (2022). 35093790 10.1016/j.tranon.2021.101334 PMC8808081

[118] Cheng, Z. et al. Tumor-derived exosomes induced M2 macrophage polarization and promoted the metastasis of osteosarcoma cells through Tim-3. *Arch. Med. Res.* **52**, 200–210 (2021). 33162186 10.1016/j.arcmed.2020.10.018

[119] Andrews, L. P. et al. Molecular pathways and mechanisms of LAG3 in cancer therapy. *Clin. Cancer Res.: Off. J. Am. Assoc. Cancer Res.* **28**, 5030–5039 (2022). 10.1158/1078-0432.CCR-21-2390 PMC9669281 35579997

[120] Aggarwal, V., Workman, C. J. & Vignali, D. A. A. LAG-3 as the third checkpoint inhibitor. *Nat. Immunol.* **24**, 1415–1422 (2023). 37488429 10.1038/s41590-023-01569-z PMC11144386

[121] Hao, Y. et al. Tumor-derived exosomes induce initial activation by exosomal CD19 antigen but impair the function of CD19-specific CAR T-cells via TGF-β signaling. *Front. Med.* (2023). 10.1007/s11684-023-1010-1 37870681

[122] Yan, G. et al. Potential impact of ALKBH5 and YTHDF1 on tumor immunity in colon adenocarcinoma. *Front. Oncol.* **11**, 670490 (2021). 34079761 10.3389/fonc.2021.670490 PMC8165310

[123] Ning, J. et al. METTL3 inhibition induced by M2 macrophage-derived extracellular vesicles drives anti-PD-1 therapy resistance via M6A-CD70-mediated immune suppression in thyroid cancer. *Cell death Differ.* **30**, 2265–2279 (2023). 37648786 10.1038/s41418-023-01217-x PMC10589295

[124] Wang, J. et al. Fibrinogen-like Protein 1 Is a Major Immune Inhibitory Ligand of LAG-3. *Cell* **176**, 334–47.e12 (2019). 30580966 10.1016/j.cell.2018.11.010 PMC6365968

[125] Maruhashi, T. et al. Binding of LAG-3 to stable peptide-MHC class II limits T cell function and suppresses autoimmunity and anti-cancer immunity. *Immunity* **55**, 912–24.e8 (2022). 35413245 10.1016/j.immuni.2022.03.013

[126] Zhang, Y. et al. FGL1 in plasma extracellular vesicles is correlated with clinical stage of lung adenocarcinoma and anti-PD-L1 response. Clinical and experimental immunology. 2023. 10.1093/cei/uxad137 PMC10929704 38146642

[127] Kouo, T. et al. Galectin-3 Shapes Antitumor Immune Responses by Suppressing CD8+ T Cells via LAG-3 and Inhibiting Expansion of Plasmacytoid Dendritic Cells. *Cancer Immunol. Res.* **3**, 412–423 (2015). 25691328 10.1158/2326-6066.CIR-14-0150 PMC4390508

[128] Yakubovich, E., Cook, D. P., Rodriguez, G. M. & Vanderhyden, B. C. Mesenchymal ovarian cancer cells promote CD8(+) T cell exhaustion through the LGALS3-LAG3 axis. *NPJ Syst. Biol. Appl.* **9**, 61 (2023). 38086828 10.1038/s41540-023-00322-4 PMC10716312

[129] Cela, I. et al. LGALS3BP is a potential target of antibody-drug conjugates in oral squamous cell carcinoma. Oral diseases. 2023. 10.1111/odi.14719 37649401

[130] Mortezaee, K. & Majidpoor, J. Alternative immune checkpoints in immunoregulatory profile of cancer stem cells. *Heliyon* **9**, e23171 (2023). 38144305 10.1016/j.heliyon.2023.e23171 PMC10746460

[131] Shi, J. et al. Molecular characteristics of single patient-derived glioma stem-like cells from primary and recurrent glioblastoma. *Anti-cancer drugs* **33**, e381–e388 (2022). 34419956 10.1097/CAD.0000000000001217 PMC8670354

[132] Solinas, C., Gu-Trantien, C., Willard-Gallo, K. The rationale behind targeting the ICOS-ICOS ligand costimulatory pathway in cancer immunotherapy. *ESMO Open*. **5** (2020). 10.1136/esmoopen-2019-000544 PMC7003380 32516116

[133] Borgeaud, M. et al. Novel targets for immune-checkpoint inhibition in cancer. *Cancer Treat. Rev.* **120**, 102614 (2023). 37603905 10.1016/j.ctrv.2023.102614

[134] Fan, X. et al. Exosome miR-27a-3p secreted from adipocytes targets ICOS to promote antitumor immunity in lung adenocarcinoma. *Thorac. cancer* **11**, 1453–1464 (2020). 32212417 10.1111/1759-7714.13411 PMC7262893

[135] Stone, E. L. et al. ICOS coreceptor signaling inactivates the transcription factor FOXO1 to promote Tfh cell differentiation. *Immunity* **42**, 239–251 (2015). 25692700 10.1016/j.immuni.2015.01.017 PMC4334393

[136] Sage, P. T., Francisco, L. M., Carman, C. V. & Sharpe, A. H. The receptor PD-1 controls follicular regulatory T cells in the lymph nodes and blood. *Nat. Immunol.* **14**, 152–161 (2013). 23242415 10.1038/ni.2496 PMC3788614

[137] Saliba, D. G. et al. Composition and structure of synaptic ectosomes exporting antigen receptor linked to functional CD40 ligand from helper T cells. eLife. 2019;8. 10.7554/eLife.47528 PMC6748831 31469364

[138] Jiang, F. et al. Extracellular vesicle-derived protein file from peripheral blood predicts immune-related adverse events in gastric cancer patients receiving immunotherapy. *Cancers*. **14** (2022). 10.3390/cancers14174167 PMC9454680 36077704

[139] Carthon, B. C. et al. Preoperative CTLA-4 blockade: tolerability and immune monitoring in the setting of a presurgical clinical trial. *Clin. Cancer Res. : Off. J. Am. Assoc. Cancer Res.* **16**, 2861–2871 (2010). 10.1158/1078-0432.CCR-10-0569 PMC2919850 20460488

[140] Koyama, S. et al. Adaptive resistance to therapeutic PD-1 blockade is associated with upregulation of alternative immune checkpoints. *Nat. Commun.* **7**, 10501 (2016). 26883990 10.1038/ncomms10501 PMC4757784

[141] Dulal, D. et al. Tackling of immunorefractory tumors by targeting alternative immune checkpoints. *Cancers* **15** (2023). 10.3390/cancers15102774 PMC10216651 37345111

[142] Lu, H. et al. B7-H3 confers resistance to Vγ9Vδ2 T cell-mediated cytotoxicity in human colon cancer cells via the STAT3/ULBP2 axis. *Cancer Immunol., Immunother. : CII* **70**, 1213–1226 (2021). 33119798 10.1007/s00262-020-02771-w PMC10992226

[143] Purvis, I. J. et al. B7-H3 in Medulloblastoma-Derived Exosomes; A Novel Tumorigenic Role. *Int. J. Mol. Sci*. **21** (2020). 10.3390/ijms21197050 PMC7583814 32992699

[144] Wu, R. et al. Exosomal B7-H3 facilitates colorectal cancer angiogenesis and metastasis through AKT1/mTOR/VEGFA pathway. *Cell. Signal.* **109**, 110737 (2023). 37263461 10.1016/j.cellsig.2023.110737

[145] Picarda, E., Ohaegbulam, K. C. & Zang, X. Molecular pathways: targeting B7-H3 (CD276) for human cancer immunotherapy. *Clin. Cancer Res: Off. J. Am. Assoc. Cancer Res.* **22**, 3425–3431 (2016). 10.1158/1078-0432.CCR-15-2428 PMC4947428 27208063

[146] Joller, N., Anderson, A. C. & Kuchroo, V. K. LAG-3, TIM-3, and TIGIT: Distinct functions in immune regulation. *Immunity* **57**, 206–222 (2024). 38354701 10.1016/j.immuni.2024.01.010 PMC10919259

[147] Swatler, J. et al. 4-1BBL-containing leukemic extracellular vesicles promote immunosuppressive effector regulatory T cells. *Blood Adv.* **6**, 1879–1894 (2022). 35130345 10.1182/bloodadvances.2021006195 PMC8941461

[148] Holmgaard, R. B. et al. Tumor-expressed IDO recruits and activates MDSCs in a Treg-dependent manner. *Cell Rep.* **13**, 412–424 (2015). 26411680 10.1016/j.celrep.2015.08.077 PMC5013825

[149] Akbar, S. et al. Circulating exosomal immuno-oncological checkpoints and cytokines are potential biomarkers to monitor tumor response to anti-PD-1/PD-L1 therapy in non-small cell lung cancer patients. *Front. Immunol.* **13**, 1097117 (2022). 36741391 10.3389/fimmu.2022.1097117 PMC9890181

[150] Jung, M. Y. et al. Superinduction of immunosuppressive glioblastoma extracellular vesicles by IFN-γ through PD-L1 and IDO1. *Neuro-Oncol. Adv.* **4**, vdac017 (2022). 10.1093/noajnl/vdac017 PMC9389426 35990703

[151] Ying, X., Zheng, X., Zhang, X., Yin, Y. & Wang, X. Kynurenine in IDO1(high) cancer cell-derived extracellular vesicles promotes angiogenesis by inducing endothelial mitophagy in ovarian cancer. *J. Transl. Med.* **22**, 267 (2024). 38468343 10.1186/s12967-024-05054-5 PMC10929174

[152] Burova, E. et al. Preclinical development of the anti-LAG-3 antibody REGN3767: characterization and activity in combination with the anti-PD-1 antibody cemiplimab in human PD-1xLAG-3-Knockin mice. *Mol. Cancer Therapeutics* **18**, 2051–2062 (2019). 10.1158/1535-7163.MCT-18-1376 31395688

[153] Euvrard, R., Robert, M., Mainbourg, S., Dalle, S., Lega, J.C. Association between immune-related adverse events and prognosis in patients treated with immune checkpoint inhibitors in melanoma: A surrogacy analysis. *Fundamental Clin. Pharmacol.* (2023). 10.1111/fcp.12966 38012082

[154] Yoo, W. S., Ku, E. J., Lee, E. K. & Ahn, H. Y. Incidence of endocrine-related dysfunction in patients treated with new immune checkpoint inhibitors: a meta-analysis and comprehensive review. *Endocrinol. Metab. (Seoul., Korea)* **38**, 750–759 (2023). 10.3803/EnM.2023.1785 PMC10764989 37956967

[155] Vardarli, I. et al. Risk and incidence of endocrine immune related adverse effects under checkpoint inhibitor mono or combination therapy in solid tumors: a meta-analysis of randomized controlled trials. The Journal of clinical endocrinology and metabolism. 2023. 10.1210/clinem/dgad670 37967245

[156] Cristescu, R. et al. Pan-tumor genomic biomarkers for PD-1 checkpoint blockade-based immunotherapy. *Science* (New York, NY). **362** (2018). 10.1126/science.aar3593 PMC6718162 30309915

[157] Rayamajhi, S. et al. Extracellular Vesicles as Liquid Biopsy Biomarkers across the Cancer Journey: From Early Detection to Recurrence. *Clin. Chem.* **70**, 206–219 (2024). 38175602 10.1093/clinchem/hvad176 PMC12374260

[158] Wang, Y. et al. Exosomal PD-L1 predicts response with immunotherapy in NSCLC patients. *Clin. Exp. Immunol.* **208**, 316–322 (2022). 35514075 10.1093/cei/uxac045 PMC9226151

[159] Eslami, S. Z. et al. Circulating tumour cells and PD-L1-positive small extracellular vesicles: the liquid biopsy combination for prognostic information in patients with metastatic non-small cell lung cancer. *Br. J. Cancer*. (2023). 10.1038/s41416-023-02491-9 PMC10781977 37973956

[160] Shen, B. et al. PD-L1 and MRN synergy in platinum-based chemoresistance of head and neck squamous cell carcinoma. *Br. J. cancer* **122**, 640–647 (2020). 31853007 10.1038/s41416-019-0697-x PMC7054324

[161] Tran, L. et al. Cisplatin Alters Antitumor Immunity and Synergizes with PD-1/PD-L1 Inhibition in Head and Neck Squamous Cell Carcinoma. *Cancer Immunol. Res.* **5**, 1141–1151 (2017). 29097421 10.1158/2326-6066.CIR-17-0235 PMC5712281

[162] Rong, Q. X. et al. GM-CSF mediates immune evasion via upregulation of PD-L1 expression in extranodal natural killer/T cell lymphoma. *Mol. cancer* **20**, 80 (2021). 34051805 10.1186/s12943-021-01374-y PMC8164269

[163] Li, J. W. et al. Universal extracellular vesicles and PD-L1+ extracellular vesicles detected by single molecule array technology as circulating biomarkers for diffuse large B cell lymphoma. *Oncoimmunology* **10**, 1995166 (2021). 34745768 10.1080/2162402X.2021.1995166 PMC8565827

[164] Zhang, Q., Jeppesen, D. K., Higginbotham, J. N., Franklin, J. L. & Coffey, R. J. Comprehensive isolation of extracellular vesicles and nanoparticles. *Nat. Protoc.* **18**, 1462–1487 (2023). 36914899 10.1038/s41596-023-00811-0 PMC10445291

[165] Gorgzadeh, A. et al. A state-of-the-art review of the recent advances in exosome isolation and detection methods in viral infection. *Virol. J.* **21**, 34 (2024). 38291452 10.1186/s12985-024-02301-5 PMC10829349

[166] Wang, C. et al. Nanoplasmonic Sandwich Immunoassay for Tumor-Derived Exosome Detection and Exosomal PD-L1 Profiling. *ACS Sens.* **6**, 3308–3319 (2021). 34494426 10.1021/acssensors.1c01101 PMC9275046

[167] Wang, Y. et al. Rapid and sensitive detection of PD-L1 exosomes using Cu-TCPP 2D MOF as a SPR sensitizer. *Biosens. Bioelectron.* **201**, 113954 (2022). 35030466 10.1016/j.bios.2021.113954

[168] Huang, M. et al. Homogeneous, Low-volume, Efficient, and Sensitive Quantitation of Circulating Exosomal PD-L1 for Cancer Diagnosis and Immunotherapy Response Prediction. *Angew. Chem. (Int. ed. Engl.)* **59**, 4800–4805 (2020). 31912940 10.1002/anie.201916039

[169] Lin, B. et al. Tracing Tumor-Derived Exosomal PD-L1 by Dual-Aptamer Activated Proximity-Induced Droplet Digital PCR. *Angew. Chem. (Int. ed. Engl.)* **60**, 7582–7586 (2021). 33382182 10.1002/anie.202015628

[170] Qin, X. et al. Simultaneous detection of cancerous exosomal miRNA-21 and PD-L1 with a sensitive dual-cycling nanoprobe. *Biosens. Bioelectron.* **216**, 114636 (2022). 35986985 10.1016/j.bios.2022.114636

[171] Chang, L. et al. Microporous PdCuB nanotag-based electrochemical aptasensor with Au@CuCl(2) nanowires interface for ultrasensitive detection of PD-L1-positive exosomes in the serum of lung cancer patients. *J. nanobiotechnology* **21**, 86 (2023). 36906540 10.1186/s12951-023-01845-y PMC10008610

[172] Pang, Y. et al. Personalized detection of circling exosomal PD-L1 based on Fe(3)O(4)@TiO(2) isolation and SERS immunoassay. *Biosens. Bioelectron.* **148**, 111800 (2020). 31678824 10.1016/j.bios.2019.111800

[173] Fan, Z. et al. Accurate and rapid quantification of PD-L1 positive exosomes by a triple-helix molecular probe. *Analytica Chim. acta* **1251**, 340984 (2023). 10.1016/j.aca.2023.340984 36925282

[174] Erman, A. et al. The prognostic and predictive value of human gastrointestinal microbiome and exosomal mRNA expression of PD-L1 and IFNγ for immune checkpoint inhibitors response in metastatic melanoma patients: PROTOCOL TRIAL. *Biomedicines* **11** (2023). 10.3390/biomedicines11072016 PMC10377397 37509655

[175] Wang, J. et al. Circulating exosomal PD-L1 at initial diagnosis predicts outcome and survival of patients with osteosarcoma. *Clin. Cancer Res.: Off. J. Am. Assoc. Cancer Res.* **29**, 659–666 (2023). 10.1158/1078-0432.CCR-22-2682 36374561

[176] Qiu, P., Guo, Q., Yao, Q., Chen, J. & Lin, J. Characterization of exosome-related gene risk model to evaluate the tumor immune microenvironment and predict prognosis in triple-negative breast cancer. *Front. Immunol.* **12**, 736030 (2021). 34659224 10.3389/fimmu.2021.736030 PMC8517454

[177] Su, D., Zhang, Z., Xu, Z., Xia, F. & Yan, Y. A prognostic exosome-related LncRNA risk model correlates with the immune microenvironment in liver cancer. *Front. Genet.* **13**, 965329 (2022). 36081999 10.3389/fgene.2022.965329 PMC9445491

[178] Gao, J. et al. Expression profiles and clinical value of plasma exosomal Tim-3 and Galectin-9 in non-small cell lung cancer. *Biochem. Biophys. Res. Commun.* **498**, 409–415 (2018). 29452091 10.1016/j.bbrc.2018.02.114

[179] Zhang, P. F. et al. Cancer cell-derived exosomal circUHRF1 induces natural killer cell exhaustion and may cause resistance to anti-PD1 therapy in hepatocellular carcinoma. *Mol. Cancer* **19**, 110 (2020). 32593303 10.1186/s12943-020-01222-5 PMC7320583

[180] Liu, L. et al. MicroRNA-15a carried by mesenchymal stem cell-derived extracellular vesicles inhibits the immune evasion of colorectal cancer cells by regulating the KDM4B/HOXC4/PD-L1 Axis. *Front. Cell Dev. Biol.* **9**, 629893 (2021). 33732698 10.3389/fcell.2021.629893 PMC7959841

[181] Chen, H. L. et al. Serum exosomal miR-16-5p functions as a tumor inhibitor and a new biomarker for PD-L1 inhibitor-dependent immunotherapy in lung adenocarcinoma by regulating PD-L1 expression. *Cancer Med.* **11**, 2627–2643 (2022). 35347894 10.1002/cam4.4638 PMC9249988

[182] Shin, J. M. et al. Sulfisoxazole elicits robust antitumour immune response along with immune checkpoint therapy by inhibiting exosomal PD-L1. *Adv. Sci. (Weinh., Baden.-Wurtt., Ger.)* **9**, e2103245 (2022). 10.1002/advs.202103245 PMC8844465 34927389

[183] Kim, G. B. et al. Harnessing oncolytic extracellular vesicles for tumor cell-preferential cytoplasmic delivery of misfolded proteins for cancer immunotherapy. *Small (Weinh. der Bergstr., Ger.)* **19**, e2300527 (2023). 10.1002/smll.202300527 37226374

[184] Im, E. J. et al. Sulfisoxazole inhibits the secretion of small extracellular vesicles by targeting the endothelin receptor A. *Nat. Commun.* **10**, 1387 (2019). 30918259 10.1038/s41467-019-09387-4 PMC6437193

[185] Lee, C. H. et al. Macitentan improves antitumor immune responses by inhibiting the secretion of tumor-derived extracellular vesicle PD-L1. *Theranostics* **12**, 1971–1987 (2022). 35265193 10.7150/thno.68864 PMC8899590

[186] Ye, H. et al. In situ sprayed nanovaccine suppressing exosomal PD-L1 by golgi apparatus disorganization for postsurgical melanoma immunotherapy. *ACS nano* **17**, 10637–10650 (2023). 37213184 10.1021/acsnano.3c01733

[187] Lallemand, T. et al. nSMase2 (Type 2-Neutral Sphingomyelinase) deficiency or inhibition by GW4869 reduces inflammation and atherosclerosis in Apoe(-/-) mice. *Arteriosclerosis, thrombosis, Vasc. Biol.* **38**, 1479–1492 (2018). 10.1161/ATVBAHA.118.311208 PMC6039418 29794115

[188] Takahashi, A. et al. Exosomes maintain cellular homeostasis by excreting harmful DNA from cells. *Nat. Commun.* **8**, 15287 (2017). 28508895 10.1038/ncomms15287 PMC5440838

[189] Deng, J. & Ke, H. Overcoming the resistance of hepatocellular carcinoma to PD-1/PD-L1 inhibitor and the resultant immunosuppression by CD38 siRNA-loaded extracellular vesicles. *Oncoimmunology* **12**, 2152635 (2023). 36605619 10.1080/2162402X.2022.2152635 PMC9809939

[190] Lee, J. & Kim, E. H. Mechanisms underlying response and resistance to immune checkpoint blockade in cancer immunotherapy. *Front. Oncol.* **13**, 1233376 (2023). 37614504 10.3389/fonc.2023.1233376 PMC10443702

[191] Chang, C. et al. Combination therapy with dendritic cell loaded-exosomes supplemented with PD-1 inhibition at different time points have superior antitumor effect in hepatocellular carcinoma. *Cancer Immunol., Immunother. CII* **72**, 3727–3738 (2023). 37665374 10.1007/s00262-023-03525-0 PMC10991982

[192] Veerman, R. E. et al. Antigen-loaded extracellular vesicles induce responsiveness to anti-PD-1 and Anti-PD-L1 treatment in a checkpoint refractory melanoma model. *Cancer Immunol. Res.* **11**, 217–227 (2023). 36546872 10.1158/2326-6066.CIR-22-0540 PMC9896027

[193] Zhang, Y. et al. Complete remission of tumors in mice with neoantigen-painted exosomes and anti-PD-1 therapy. *Mol. Ther.: J. Am. Soc. Gene Ther.* **31**, 3579–3593 (2023). 10.1016/j.ymthe.2023.10.021 PMC10727972 37919900

[194] Cifuentes-Rius, A., Desai, A., Yuen, D., Johnston, A. P. R. & Voelcker, N. H. Inducing immune tolerance with dendritic cell-targeting nanomedicines. *Nat. Nanotechnol.* **16**, 37–46 (2021). 33349685 10.1038/s41565-020-00810-2

[195] Phung, C. D. et al. Anti-CTLA-4 antibody-functionalized dendritic cell-derived exosomes targeting tumor-draining lymph nodes for effective induction of antitumor T-cell responses. *Acta Biomater.* **115**, 371–382 (2020). 32798721 10.1016/j.actbio.2020.08.008

[196] Liu, Y., Sun, L., Li, Y. & Holmes, C. Mesenchymal stromal/stem cell tissue source and in vitro expansion impact extracellular vesicle protein and miRNA compositions as well as angiogenic and immunomodulatory capacities. *J. Extracell. Vesicles* **13**, e12472 (2024). 39092563 10.1002/jev2.12472 PMC11294870

[197] Zhao, L. et al. LOXL4 shuttled by tumor cells-derived extracellular vesicles promotes immune escape in hepatocellular carcinoma by activating the STAT1/PD-L1 Axis. *Journal of immunotherapy (Hagerstown, Md : 1997)* (2023). 10.1097/CJI.0000000000000496 38047403

[198] Zheng, Y. et al. Identification of extracellular vesicles-transported miRNAs in Erlotinib-resistant head and neck squamous cell carcinoma. *J. cell Commun. Signal.* **14**, 389–402 (2020). 32157550 10.1007/s12079-020-00546-7 PMC7642164

[199] Yin, Y. et al. Colorectal cancer-derived small extracellular vesicles promote tumor immune evasion by upregulating PD-L1 expression in tumor-associated macrophages. *Adv. Sci. (Weinh., Baden.-Wurtt., Ger.)* **9**, 2102620 (2022). 10.1002/advs.202102620 PMC8948581 35356153

[200] Rabe, D. C. et al. Tumor extracellular vesicles regulate macrophage-driven metastasis through CCL5. *Cancers* **13** (2021). 10.3390/cancers13143459 PMC8303898 34298673

[201] Zhang, C. et al. Anti-PD-1 therapy response predicted by the combination of exosomal PD-L1 and CD28. *Front. Oncol.* **10**, 760 (2020). 32528882 10.3389/fonc.2020.00760 PMC7266952

[202] Shimada, Y. et al. Serum-derived exosomal PD-L1 expression to predict anti-PD-1 response and in patients with non-small cell lung cancer. *Sci. Rep.* **11**, 7830 (2021). 33837261 10.1038/s41598-021-87575-3 PMC8035184

[203] Hao, J. et al. Homogeneous, simple, and direct analysis of exosomal PD-L1 via aptamer-bivalent-cholesterol-anchor assembly of DNAzyme (ABCzyme) for tumor immunotherapy. *Anal. Chem.* **95**, 6854–6862 (2023). 37027485 10.1021/acs.analchem.2c05461

