# Author Correction: Influencing immunity: role of extracellular vesicles in tumor immune checkpoint dynamics

**DOI:** 10.1038/s12276-025-01394-4

**Published:** 2025-01-20

**Authors:** Ziyang Ye, Genpeng Li, Jianyong Lei

**Affiliations:** Division of Thyroid Surgery, Department of General Surgery, West China Hospital, Sichuan University, Chengdu, China

**Keywords:** Immunoediting, Tumour biomarkers

Correction to: *Experimental & Molecular Medicine* 10.1038/s12276-024-01340-w, published online 11 November 2024

In the Funding section of this article the grant number relating to the National Natural Science Foundation of China was incorrectly given as 82173245, 82300881, 82473062 and should have been 82473062, 82173245, 82300881.

The correct statement of this article should have read as below:

This study was supported by grants from the National Natural Science Foundation of China (82473062, 82173245, 82300881); the Sichuan Science and Technology Program (2023YFS0148 and 2023YFS0149); the Fundamental Research Funds for the Central Universities (2022SCU12061); and the postdoctoral project, West China Hospital, Sichuan University (2023HXBH051).

The original article has been corrected.

